# Increasing national trend of direct-acting antiviral discontinuation among people treated for HCV 2016–2021

**DOI:** 10.1097/HC9.0000000000000125

**Published:** 2023-03-30

**Authors:** Joanne Carson, Sebastiano Barbieri, Gail V. Matthews, Gregory J. Dore, Behzad Hajarizadeh

**Affiliations:** 1 The Kirby Institute, UNSW Sydney, Sydney, New South Wales, Australia; 2 Centre for Big Data Research in Health, UNSW Sydney, Sydney, New South Wales, Australia

## Abstract

Direct-acting antiviral (DAA) treatment discontinuation may negatively impact HCV elimination efforts. In Australia, DAA therapy is pharmacy dispensed, generally in 4-week amounts, with the approved duration (8–24 wk) and volume dispensed reported in pharmaceutical administrative data. This analysis assessed national HCV treatment discontinuation.

**Methods:**

Individuals commencing DAAs between 2016 and 2021 were assessed for treatment discontinuation. Individuals with a single dispensation of their entire treatment course were excluded. Treatment discontinuation was defined as ≥4 weeks of approved treatment duration not dispensed. Factors associated with treatment discontinuation were assessed using Cox regression. Factors associated with retreatment following treatment discontinuation were assessed using logistic regression.

**Results:**

Of 95,275 individuals who were treated, 88,986 were included in the analysis of whom 7532 (9%) discontinued treatment. Treatment discontinuation increased from 6% in the first half of 2016 to 15% in 2021. Longer treatment durations (vs. 8 wk) were associated with increased discontinuation risk (12 wk: adjusted HR = 3.23; 95% CI: 2.90, 3.59; *p* < 0.001, 16–24 wk: adjusted HR = 6.29; 95% CI: 5.55, 7.14; *p* < 0.001). Of individuals discontinuing treatment, 24% were retreated. Early discontinuation (4 wk treatment dispensed) increased the likelihood of retreatment (adjusted OR = 3.91; 95% CI: 3.44, 4.44; *p* < 0.001). Those with early discontinuation of glecaprevir/pibrentasvir 8 weeks (vs. sofosbuvir/velpatasvir 12 wk) had a lower likelihood of retreatment (adjusted OR = 0.62; 95% CI: 0.49, 0.79; *p* < 0.001). Initial treatment discontinuation was associated with an increased risk of retreatment discontinuation (adjusted HR = 4.41; 3.85, 5.05; *p* < 0.001).

**Conclusions:**

DAA treatment discontinuation increased over time corresponding to increasing treatment uptake through primary care among people who inject drugs. The use of simplified, short-duration therapies may reduce treatment discontinuation. Access to adherence support and retreatment will be essential for HCV elimination.

## Graphical abstract

**Figure:**
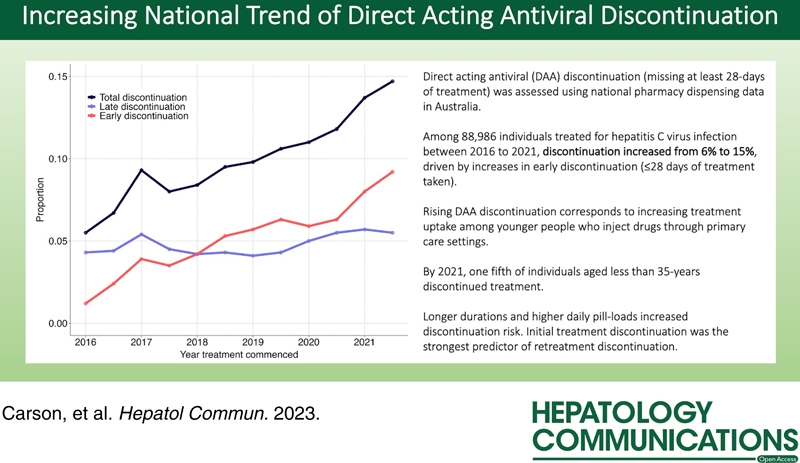

## INTRODUCTION

Treatment for HCV infection has evolved considerably in the past decade. The development of tolerable, short-duration direct-acting antiviral (DAA) therapies that are easy to administer and highly effective compared with interferon-based predecessors led to an almost 10-fold increase in the global uptake of treatment between 2015 and 2019.^1^,^2^ Historically, people who inject drugs were not prioritized for treatment,^3^ however, now treating those with the highest risk of HCV transmission is considered a central tenet of efforts to eliminate HCV as a major public health threat by 2030.^1^,^3^

Despite the advances of DAA therapies, treatment discontinuation has emerged as a major reason for treatment failure. In a recent analysis assessing the national uptake of retreatment in Australia, half of those retreated for treatment failure had discontinued initial treatment.^4^ In Australia, treatment patterns have changed over time, with older individuals with advanced liver disease initially prioritized, and subsequently broadening to younger populations, including people who inject drugs.^5^,^6^

The primary aim of this analysis is to assess DAA treatment and retreatment discontinuation over time at a national level. The secondary aim is to identify factors associated with DAA discontinuation and the factors associated with retreatment among those discontinuing initial treatment.

## METHODS

### Study setting and data sources

The Australian Pharmaceutical Benefit Scheme (PBS) is a government program that subsidizes prescription medicines, including DAAs. The PBS is available to all Australian citizens, permanent residents, and persons from countries with whom Australia has a reciprocal health care arrangement.^7^ DAAs were made available through the PBS in March 2016 through an unrestricted access scheme, with no limitations on treatment or retreatment.^5^ The approved durations of these regimens range from 8 to 24 weeks depending on DAA type and the clinical characteristics of the patient (Supplemental Table 1, http://links.lww.com/HC9/A231). DAA treatment is typically dispensed in 28-day packages (4 wk supply at a time), standard DAA dosage ranged from 1 to 4 pills daily (Supplemental Table 1, http://links.lww.com/HC9/A231). Currently under the PBS, the cost of all listed medications, including DAAs, is AUD$30 for a 28-day supply, decreasing to AUD$7 per 28-day supply among those eligible for low-income subsidy prescriptions.^7^ Low-income subsidy eligibility includes those who are unemployed, those receiving disability support,

The process of prescribing DAAs is the same for all medical practitioners in Australia and includes calling the PBS, supplying details of patient eligibility, and obtaining an authorization code for the prescription (a process that typically takes <5 min).^8^ The prescription is provided to the patient, in paper or electronic form (through SMS), with a specified number of repeats that can be filled at any community or hospital pharmacy within Australia. There is no requirement for on-treatment blood tests to assess response to DAA therapy or for the patient to be abstinent from alcohol or drugs during treatment.^9^

All pharmacy dispensation of DAAs, including initial supply and the filling of repeat prescriptions are reported through PBS. These administrative records provide individual-level data of prescribed medications (name, dosage, and duration), dates of prescription and dispensing, number of doses dispensed, patient’s age, sex, and residence area, and prescriber speciality. All instances of treatment and retreatment are collected by PBS. National PBS data were available from March 2016 to June 2022. As individuals may not commence treatment immediately following initial dispensation, those commencing treatment or retreatment between March 2016 and December 2021 were followed until June 2022 to assess discontinuation.

### Study definitions

As there is limited clinical detail in the PBS data, specific clinical definitions were used for the analysis of PBS data. The end of treatment for each prescription was estimated based on the known approved duration of the DAA regimen. Treatment discontinuation was defined as 1 or more repeat authorized prescription courses (28-d supply) not dispensed. Early discontinuation was defined as discontinuing after the first 28 days of the treatment course had been dispensed. Late discontinuation was defined as discontinuing after 56 days or more had been dispensed. Individuals who discontinued initial treatment and restarted a different regimen ≤28 days before the estimated end of treatment were considered treatment switches and were not considered treatment discontinuations or retreatments. Retreatment was defined as the commencement of a different DAA regimen any time after the end of the initial treatment date or the same DAA regimen ≥28 days after the last dispensation of the initial treatment, to allow for prescribers extending the duration of therapy due to nonadherence. Individuals who were dispensed antiretroviral therapy regimens were defined as individuals with HIV coinfection. Prescriber caseloads were defined as low (prescribed DAAs to <10 individuals), medium (prescribed DAAs to 10–100 individuals), or high (prescribed DAAs to >100 individuals). COVID-19 pandemic restrictions were considered as occurring between March 2020 and September 2021, although restrictions occurred intermittently in Australian jurisdictions during this period. Remoteness was classified according to the home post-code of the patient using the Australian Geography Standard Remoteness Structure.^10^

### Statistical analysis

Individuals who received their entire treatment course in a single dispensation could not be assessed for treatment discontinuation and were excluded from the analysis. In the analysis population, the estimated number and proportion of individuals who discontinued treatment between 2016 and 2021 were calculated and plotted against the year of treatment commencement. Interrupted time series regression analysis was used to assess the impact of COVID-19 restrictions on treatment discontinuation with adjustment for calendar time (1-mo intervals).^11^ Cox proportional hazards regression using dosage dispensation count as the time variable was used to assess factors associated with time to treatment discontinuation. Individuals remained at risk of discontinuation for the authorized duration of their prescribed treatment course. Those that completed the authorized duration of treatment were censored at this point. Covariates included in the model were sex, age, HIV coinfection, remoteness, DAA regimen, DAA duration, year DAA regimen commenced, prescription cost (general or low-income subsidy), prescriber type, and prescriber caseload. For the analysis assessing factors associated with retreatment discontinuation, previous treatment discontinuation was also included as a covariate. The *R* ^2^ value was used to quantify the amount of variation in treatment discontinuation that was explained by the factors included in the model (goodness of fit).^10^ Higher values indicate that more variation is accounted for by the model.

As sofosbuvir/velpatsvir (SOF/VEL) 12 weeks and glecaprevir/pibrentasvir (GLE/PIB) 8 weeks accounted for the majority (88%) of DAA prescriptions between 2018 and 2021, an additional Cox regression analysis restricted to these DAA regimens and time-period was conducted to assess treatment discontinuation.

Logistic regression was used to assess factors associated with retreatment among those with treatment discontinuation. Additional logistic regression analysis restricted to those with treatment discontinuation of SOF/VEL 12 weeks or GLE-PIB 8 weeks was conducted to assess factors associated with retreatment.

In all regression models, clinically plausible covariates with a *p*-value < 0.20 in unadjusted models were included in the adjusted model. Statistical significance was assessed at *p*-value < 0.05 (2-sided *p*-values). Statistical analyses were performed in R, version 2.4.0 (using survival, survmetrics, coxR2, glm, its.analysis packages).

## RESULTS

### Characteristics of the study population

A total of 95,275 individuals commenced treatment between 2016 and 2021 (Supplemental Figures 1 and 2, http://links.lww.com/HC9/A231). Table 1 presents the characteristics of the 88,986 individuals who were included in the assessment of treatment discontinuation. The characteristics of 6289 individuals who received their entire treatment course in a single dispensation and were excluded from this analysis are displayed in Supplemental Table 2 (http://links.lww.com/HC9/A231). In the analysis population, the median age was 49 years (interquartile range: 40, 57), most were male (67%), resided in major cities (65%), and received low-income subsidy prescriptions (61%). Changes in DAA prescribing patterns and the population receiving treatment were observed between 2016 and 2021. In the first half of 2016, the most common prescribers were gastroenterologists (55%) and the most common regimens prescribed were sofosbuvir/ledipasvir (SOF/LDV; 58%) and sofosbuvir/daclatasvir (SOF+DCV; 37%). By the second half of 2021, SOF/VEL (54%) and GLE/PIB (45%) were the dominant regimens, and general practitioners were the majority prescribers (55%). Of the 10,543 unique prescribers of DAA therapy in Australia, most were general practitioners (87%) and the median number of individuals treated per prescriber was 2 (interquartile range: 1, 4) with a range of 1–1572. An assessment of prescriber caseload in relation to time prescribing DAAs can be found in Supplemental Figure 3 (http://links.lww.com/HC9/A231).

**TABLE 1:** Characteristics of the study population and factors associated with treatment discontinuation

	n (%)						
	Treated (n = 88,986)	Treatment fully dispensed (n = 81,454)	Treatment discontinued (n = 7532)	Unadjusted HR (95% CI)	*p*	Adjusted HR (95% CI)	*p*
Age [median (IQR)] (y)	49 (40, 57)	49 (40, 57)	45 (36, 54)	1.29 (1.27, 1.32)^a^	<0.001	1.29 (1.27, 1.32)^a^	<0.001
Sex							
Male	59,852 (67.3)	54,891 (67.4)	4961 (65.9)	—	—	—	—
Female	29,134 (32.7)	26,563 (32.6)	2571 (34.1)	1.09 (1.04, 1.14)	0.001	1.07 (1.02, 1.13)	0.004
Concurrent treatment for HIV							
No	87,442 (98.3)	80,043 (98.3)	7399 (98.2)	—	—		
Yes	1544 (1.7)	1411 (1.7)	133 (1.8)	1.01 (0.85, 1.19)	0.943		
Location of patient residence							
Major city	57,484 (64.6)	52,666 (64.7)	4818 (64.0)	—	—	—	—
Regional	28,646 (32.2)	26,188 (32.2)	2458 (32.6)	1.03 (0.98, 1.08)	0.271	0.97 (0.92, 1.02)	0.243
Remote	1352 (1.5)	1205 (1.5)	147 (2.0)	1.32 (1.12, 1.55)	0.001	1.26 (1.07, 1.49)	0.006
Unavailable	1504 (1.7)	1395 (1.7)	109 (1.4)	0.86 (0.71, 1.04)	0.117	0.76 (0.63, 0.92)	0.006
Prescription payment type							
General	34,917 (39.2)	32,705 (40.2)	2212 (29.4)	—	—	—	—
Low-income subsidy	54,069 (60.8)	48,749 (59.8)	5320 (70.6)	1.56 (1.49, 1.64)	<0.001	1.50 (1.43, 1.58)	<0.001
Year treatment commenced							
2016	32,274 (36.3)	30,321 (37.2)	1953 (25.9)	—	—	—	—
2017	20,861 (23.4)	19,052 (23.4)	1809 (24.0)	1.65 (1.55, 1.76)	<0.001	1.52 (1.41, 1.63)	<0.001
2018	13,851 (15.6)	12,614 (15.5)	1237 (16.4)	1.84 (1.71, 1.98)	<0.001	1.81 (1.64, 2.01)	<0.001
2019	9905 (11.1)	8897 (10.9)	1008 (13.4)	2.16 (2.00, 2.33)	<0.001	2.15 (1.92, 2.41)	<0.001
2020	6746 (7.6)	5980 (7.3)	766 (10.2)	2.43 (2.23, 2.64)	<0.001	2.35 (2.08, 2.65)	<0.001
2021	5349 (6.0)	4590 (5.6)	759 (10.1)	3.11 (2.86, 3.39)	<0.001	3.14 (2.77, 3.55)	<0.001
Prescriber type							
Gastroenterologist	32,455 (36.5)	30,373 (37.3)	2082 (27.6)	—	—	—	—
Infectious diseases physician	5477 (6.2)	5037 (6.2)	440 (5.8)	1.34 (1.21, 1.49)	<0.001	1.27 (1.14, 1.41)	<0.001
General practitioner	39,752 (44.7)	35,663 (43.8)	4089 (54.3)	1.80 (1.71, 1.90)	<0.001	1.41 (1.32, 1.50)	<0.001
General physician	6022 (6.8)	5614 (6.9)	408 (5.4)	1.05 (0.94, 1.17)	0.364	1.05 (0.94, 1.17)	0.357
Sexual physician	1242 (1.4)	1117 (1.4)	125 (1.7)	1.79 (1.46, 2.09)	<0.001	1.69 (1.41, 2.03)	<0.001
Psychiatry or addiction specialist	1027 (1.2)	920 (1.1)	107 (1.4)	1.87 (1.54, 2.27)	<0.001	1.26 (1.03, 1.53)	0.022
Nurse practitioner	978 (1.1)	873 (1.1)	105 (1.4)	1.97 (1.51, 1.62)	<0.001	1.11 (0.91, 1.35)	0.315
Other specialist	1282 (1.4)	1164 (1.4)	118 (1.6)	1.51 (1.25, 1.81)	<0.001	1.18 (0.98, 1.43)	0.078
Unavailable	751 (0.8)	693 (0.9)	58 (0.8)	1.40 (1.08, 1.82)	<0.001	0.96 (0.73, 1.25)	0.744
Number of patients per prescriber							
High (≥100 patients)	41,583 (46.7)	38,494 (47.3)	3089 (41.0)	—	—	—	—
Medium (10–100 patients)	29,059 (32.7)	26,636 (32.7)	2423 (32.2)	1.14 (1.08, 1.20)	<0.001	0.99 (0.93, 1.05)	0.665
Low (<10 patients)	18,344 (20.6)	16,324 (20.0)	2020 (26.8)	1.65 (1.56, 1.75)	<0.001	1.08 (1.01, 1.15)	0.037
DAA regimen							
SOF/VEL	26,218 (29.5)	23,794 (29.2)	2424 (32.2)	—	—	—	—
SOF/LDV	27,046 (30.4)	25,470 (31.3)	1576 (20.9)	0.58 (0.54, 0.61)	<0.001	1.22 (1.11, 1.34)	<0.001
SOF+DCV	18,686 (21.0)	17,039 (20.9)	1647 (21.9)	0.72 (0.68, 0.77)	<0.001	1.10 (1.00, 1.21)	0.061
SOF+RBV	1883 (2.1)	1775 (2.2)	108 (1.4)	0.57 (0.47, 0.69)	<0.001	1.16 (0.91, 1.49)	0.234
PrOD	588 (0.7)	504 (0.6)	84 (1.1)	1.54 (1.24, 1.92)	<0.001	3.45 (2.74, 4.33)	<0.001
GRZ/ELB	4190 (4.7)	3714 (4.6)	476 (6.3)	1.23 (1.11, 1.36)	<0.001	1.43 (1.29, 1.59)	<0.001
GLE/PIB	10,271 (11.5)	9069 (11.1)	1202 (16.0)	1.39 (1.30, 1.49)	<0.001	2.84 (2.56, 3.14)	<0.001
Other^b^	104 (0.1)	89 (0.1)	15 (0.2)	1.49 (0.89, 2.48)	0.122	1.73 (1.04, 2.88)	0.034
Ribavirin added to DAA regimen							
No	86,291 (97.0)	78,951 (96.9)	7340 (97.5)	—	—	—	—
Yes	2695 (3.0)	2503 (3.1)	192 (2.5)	0.78 (0.68, 0.91)	<0.001	1.02 (0.85, 1.22)	0.815
Treatment duration							
8 wk	13,798 (15.5)	12,854 (15.8)	944 (12.5)	—	—	—	—
12 wk	66,716 (75.0)	61,414 (75.4)	5302 (70.4)	1.06 (0.99, 1.13)	0.124	3.23 (2.90, 3.59)	<0.001
16 or 24 wk	8472 (9.5)	7186 (8.8)	1286 (17.1)	1.23 (1.12, 1.36)	<0.001	6.29 (5.55, 7.14)	<0.001

^a^ HR calculated per 10-year decrease in age.

^b^ Other includes glecaprevir/pibrentasvir+sofosbuvir; grazoprevir/elbasvir+sofosbuvir, paritaprevir/ritonavir/ombitasvir+dasabuvir+sofosbuvir, sofosbuvir/ledipasvir+daclatasvir.

Abbreviations: DAA, direct-acting antiviral; GLE/PIB, glecaprevir/pibrentasvir; GRZ/ELB, grazoprevir/elbasvir; IQR, interquartile range; PrOD, paritaprevir/ritonavir/ombitasvir+dasabuvir; SOF+DCV, sofosbuvir+daclatasvir; SOF/LDV, sofosbuvir/ledipasvir; SOF+RBV, sofosbuvir+ribavirin±interferon; SOF/VEL; sofosbuvir/velpatasvir.

### Treatment discontinuation

**FIGURE 1:**
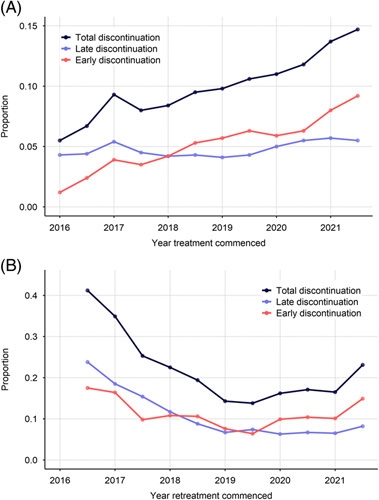
Proportion of treatment and retreatment discontinuation between 2016 and 2021. (A) Early and late treatment discontinuation. (B) Early and late retreatment discontinuation.

Table 1 presents factors associated with treatment discontinuation. Figure 1 presents treatment discontinuation over time. Overall, 9% (n = 7532) of those treated discontinued treatment, increasing from 6% in the first half of 2016 to 15% in the second half of 2021. Early treatment discontinuation (only 28 d dispensed) increased from 1% in the first half of 2016 to 9% in the second half of 2021. Late discontinuation remained relatively stable (average = 5%). Between the first half of 2016 and the second half of 2021, treatment discontinuation increased from 8% to 19% among those aged less than 35 years; from 6% to 17% among females; from 6% to 18% among those treated by general practitioners and from 6% to 17% among those treated by low-caseload prescribers.

Treatment discontinuation was 7% for those prescribed 8-week durations, 8% for those prescribed 12-week durations, and 15% for those prescribed 16- to 24-week durations.

In interrupted time series analysis, no significant impact of COVID-19 restrictions on the trend of treatment discontinuation was observed (Supplemental Figure 4, http://links.lww.com/HC9/A231).

**FIGURE 2:**
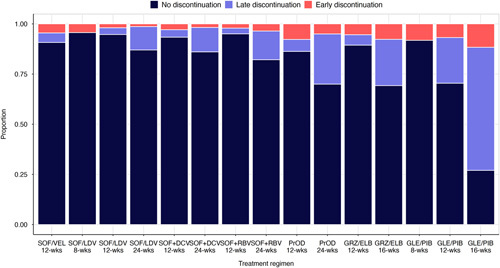
Proportion of early and late treatment discontinuation by treatment regimen and duration. Abbreviations: GLE/PIB, glecaprevir/pibrentasvir; GRZ/ELB, grazoprevir/elbasvir; PrOD, paritaprevir/ritonavir/ombitasvir+dasabuvir; SOF+DCV, sofosbuvir+daclatasvir; SOF/LDV, sofosbuvir/ledipasvir; SOF+RBV, sofosbuvir+ribavirin±interferon; SOF/VEL; sofosbuvir/velpatasvir.

In adjusted Cox regression analysis, longer treatment durations, DAA regimens with higher daily pill loads, and later year of treatment commencement were associated with the greatest risk of treatment discontinuation. Compared with individuals prescribed 8 weeks treatment duration, those prescribed 12 weeks (adjusted HR = 3.23; 95% CI: 2.90, 3.59; *p* < 0.001) and 16–24 weeks (adjusted HR = 6.29; 95% CI: 5.55, 7.14; *p* < 0.001) treatment durations had increased discontinuation risk. Treatment regimens associated with the greatest discontinuation risk (vs. SOF/VEL) were paritaprevir/ritonavir/ombitasvir+dasabuvir (PrOD; adjusted HR = 3.45; 95% CI: 2.74, 4.33; *p* < 0.001) and GLE/PIB (adjusted HR = 2.84; 95% CI: 2.56, 3.14; *p* < 0.001). Of note, PrOD (4 pills daily) and GLE/PIB (3 pills daily) have higher daily pill loads than other DAA regimens. Figure 2 displays early and late discontinuation rates for specific treatment regimens and durations. A trend of increasing discontinuation risk among individuals treated in later years was observed, with the greatest risk among those commencing treatment in 2021 (vs. 2016: adjusted HR = 3.14; 95% CI: 2.77, 3.55; *p* < 0.001). Other factors associated with treatment discontinuation included younger age, female sex, receiving low-income subsidy prescriptions, residing in a remote location, and treatment by the low-caseload prescriber. Prescriber types associated with increased discontinuation risk were general practitioners, sexual health physicians, infectious diseases physicians, and psychiatry or addition specialists (vs. gastroenterologists). The *R* ^2^ value for the treatment discontinuation model was 0.32.

When analysis of treatment discontinuation was restricted to those receiving GLE/PIB 8 weeks or SOF/VEL 12 weeks between 2018 and 2021 (n = 29,984), a lower risk of discontinuation was observed for GLE/PIB 8 weeks (adjusted HR = 0.75; 95% CI: 0.68, 0.81, *p* < 0.001; Table 2). Other factors remained consistent with the original model.

**TABLE 2:** Factors associated with treatment discontinuation of SOF/VEL 12 weeks and GLE/PIB 8 weeks

	n (%)					
	Treatment fully dispensed (n = 27,205)	Treatment discontinued (n = 2779)	Unadjusted HR (95% CI)	*p*	Adjusted HR (95% CI)	*p*
Age [median (IQR)] (y)	47 (38, 56)	43 (35, 51)	1.26 (1.22, 1.29)^a^	<0.001	1.31 (1.27, 1.35)^a^	<0.001
Sex						
Male	18,690 (68.7)	1756 (63.2)	—			
Female	8515 (31.3)	1023 (36.8)	1.27 (1.17, 1.37)	<0.001	1.15 (1.07, 1.25)	<0.001
Concurrent treatment for HIV						
No	26,941 (99.0)	2731 (98.3)	—	—	—	—
Yes	264 (1.0)	48 (1.7)	1.72 (1.29, 2.28)	<0.001	1.68 (1.26, 2.25)	<0.001
Location of patient residence						
Major city	17,466 (64.2)	1809 (65.1)	—	—	—	—
Regional	8778 (32.3)	863 (31.1)	0.95 (0.88, 1.03)	0.214	0.90 (0.83, 0.98)	0.016
Remote	415 (1.5)	68 (2.4)	1.54 (1.21, 1.96)	<0.001	1.50 (1.18, 1.92)	0.001
Unavailable	546 (2.0)	39 (1.4)	0.69 (0.51, 0.96)	0.026	0.65 (0.47, 0.89)	0.008
Prescription payment type						
General	10,696 (39.3)	657 (23.6)	—	—	—	—
Low-income subsidy	16,509 (60.7)	2122 (76.4)	2.04 (1.87, 2.23)	<0.001	1.95 (1.78, 2.14)	<0.001
Year treatment commenced						
2018	9633 (35.4)	816 (29.4)	—	—	—	—
2019	7715 (28.4)	775 (27.9)	1.18 (1.07, 1.30)	0.001	1.25 (1.13, 1.39)	<0.001
2020	5473 (20.1)	569 (20.5)	1.22 (1.10, 1.36)	<0.001	1.26 (1.13, 1.41)	<0.001
2021	4384 (16.1)	619 (22.3)	1.63 (1.47, 1.81)	<0.001	1.73 (1.55, 1.93)	<0.001
Prescriber type						
Gastroenterologist	7657 (28.1)	521 (18.7)	—	—	—	—
Infectious diseases physician	1724 (6.3)	142 (5.1)	1.20 (1.00, 1.45)	0.051	1.12 (0.93, 1.35)	0.239
General practitioner	14,093 (51.8)	1756 (63.2)	1.81 (1.64, 1.99)	0.034	1.36 (1.21, 1.53)	<0.001
General physician	1134 (4.2)	99 (3.6)	1.26 (1.02, 1.56)	<0.001	1.17 (0.94, 1.45)	0.157
Sexual physician	380 (1.4)	47 (1.7)	1.79 (1.33, 2.42)	<0.001	1.55 (1.14, 2.10)	0.005
Psychiatry or addiction specialist	466 (1.7)	54 (1.9)	1.68 (1.27, 2.22)	<0.001	1.15 (0.86, 1.53)	0.342
Nurse practitioner	755 (2.8)	85 (3.1)	1.62 (1.29, 2.04)	<0.001	1.05 (0.83, 1.32)	0.696
Other specialist	354 (1.3)	30 (1.1)	1.23 (0.85, 1.77)	0.275	0.84 (0.58, 1.21)	0.344
Unavailable	642 (2.4)	45 (1.6)	1.03 (0.76, 1.40)	0.856	0.85 (0.63, 1.16)	0.310
Number of patients per prescriber						
High (≥100 patients)	11,712 (43.1)	942 (33.9)	—	—	—	—
Medium (10–100 patients)	8004 (29.4)	816 (29.4)	1.25 (1.14, 1.38)	<0.001	1.01 (0.92, 1.12)	0.790
Low (<10 patients)	7489 (27.5)	1021 (36.7)	1.69 (1.54, 1.84)	<0.001	1.16 (1.04, 1.30)	0.008
DAA regimen						
SOF/VEL 12 wk	19,016 (69.9)	2047 (73.7)	—	—	—	—
GLE/PIB 8 wk	8189 (30.1)	732 (26.3)	0.86 (0.79, 0.93)	<0.001	0.75 (0.68, 0.81)	<0.001

^a^ HR calculated per 10-year decrease in age.

Abbreviations: DAA, direct-acting antiviral; GLE/PIB, glecepravir/pibrentasvir; IQR, interquartile range; SOF/VEL, sofosbuvir/velpatasvir.

When factors associated with early discontinuation of treatment (at 4 wk) were assessed (Supplemental Table 3, http://links.lww.com/HC9/A231), individuals prescribed longer durations of treatment had a lower likelihood of early discontinuation. Compared with individuals prescribed 8 weeks treatment duration, those prescribed 12 weeks (adjusted OR = 0.72; 95% CI: 0.63, 0.84; *p* < 0.001) and 16–24 weeks (adjusted OR = 0.74; 95% CI: 0.59, 0.84; *p* < 0.001) treatment durations had reduced likelihood of early discontinuation. Other factors remained consistent with the original model. The pseudo-*R* ^2^ value for early discontinuation was 0.07.

### Retreatment following treatment discontinuation

Table 3 displays factors associated with retreatment among those who discontinued initial treatment. Of individuals discontinuing treatment (n = 7532), 24% were retreated. Early treatment discontinuation (vs. late discontinuation; adjusted HR = 3.91; 95% CI: 3.44, 4.44; *p* < 0.001) was associated with increased likelihood of retreatment. Treatment regimens associated with increased likelihood of retreatment (vs. SOF/VEL) included sofosbuvir+ribavirin (SOF+RBV; adjusted HR = 3.81; 95% 2.18, 6.72; <0.001) or SOF+DCV (adjusted HR = 1.34; 95% CI: 1.07, 1.68; *p* = 0.010). Treatment regimens with a decreased likelihood of retreatment following discontinuation were GLE/PIB (adjusted HR=0.59; 95% CI: 0.45, 0.77; *p* < 0.001) and grazoprevir/elbasvir (GRZ/ELB; adjusted HR = 0.60; 95% CI: 0.45, 0.78; *p* < 0.001). Other factors associated with a decreased likelihood of retreatment included female sex (adjusted OR = 0.74; 95% CI: 0.65, 0.83; *p* < 0.001), and treatment by low-caseload prescriber (adjusted OR = 0.70; 95% CI: 0.59, 0.83, *p* < 0.001).

**TABLE 3:** Factors associated with retreatment among people who discontinued initial treatment

	n (%)					
	Not retreated (n = 5697)	Retreated (n = 1835)	Unadjusted OR (95% CI)	*p*	Adjusted OR (95% CI)	*p*
Age [median (IQR)] (y)	46 (37, 55)	43 (34, 52)	1.28 (1.16, 1.26)^b^	<0.001	1.17 (1.12, 1.23)^b^	<0.001
Sex						
Male	3681 (64.6)	1280 (69.8)	—	—	—	—
Female	2016 (35.4)	555 (30.2)	0.79 (0.71, 0.89)	<0.001	0.74 (0.65, 0.83)	<0.001
Concurrent treatment for HIV						
No	5605 (98.4)	1794 (97.8)	—	—	—	—
Yes	92 (1.6)	41 (2.2)	1.39 (0.95, 2.00)	0.081	1.35 (0.89, 2.01)	0.149
Location of patient residence						
Major city	3609 (63.3)	1209 (65.9)	—	—	—	—
Regional	1903 (33.4)	555 (30.2)	0.87 (0.78, 0.98)	0.018	0.90 (0.79, 1.02)	0.096
Remote	106 (1.9)	41 (2.2)	1.15 (0.79, 1.65)	0.442	1.23 (0.82, 1.82)	0.299
Unavailable	79 (1.4)	30 (1.6)	1.13 (0.73, 1.71)	0.563	1.35 (0.84, 2.14)	0.207
Prescription payment type						
General	1696 (29.8)	516 (28.1)	—	—	—	—
Low-income subsidy	4001 (70.2)	1319 (71.9)	1.08 (0.96, 1.22)	0.177	1.07 (0.94, 1.22)	0.304
Year treatment commenced						
2016	1503 (26.4)	450 (24.5)	—	—	—	—
2017	1314 (23.1)	495 (27.0)	1.26 (1.09, 1.46)	0.002	1.04 (0.87, 1.24)	0.652
2018	845 (14.8)	392 (21.4)	1.55 (1.32, 1.82)	<0.001	1.23 (0.97, 1.55)	0.081
2019	738 (13.0)	270 (14.7)	1.22 (1.02, 1.45)	0.025	1.00 (0.77, 1.31)	0.989
2020	617 (10.8)	149 (8.1)	0.81 (0.65, 0.99)	0.042	0.74 (0.55, 0.99)	0.044
2021	680 (11.9)	79 (4.3)	0.39 (0.30, 0.50)	<0.001	0.31 (0.22, 0.43)	<0.001
Prescriber type						
Gastroenterologist	1619 (28.4)	463 (25.2)	—	—	—	—
Infectious diseases physician	343 (6.0)	97 (5.3)	0.99 (0.77, 1.26)	0.930	0.88 (0.67, 1.15)	0.366
General practitioner	3017 (53.0)	1072 (58.4)	1.24 (1.10, 1.41)	<0.001	1.22 (1.04, 1.42)	0.014
General physician	326 (5.7)	82 (4.5)	0.88 (0.67, 1.14)	0.339	0.84 (0.64, 1.11)	0.239
Sexual physician	90 (1.6)	35 (1.9)	1.36 (0.90, 2.02)	0.136	1.08 (0.69, 1.65)	0.737
Psychiatry or addiction specialist	74 (1.3)	33 (1.8)	1.56 (1.01, 2.02)	0.039	1.38 (0.86, 2.18)	0.171
Nurse practitioner	90 (1.6)	15 (0.8)	0.58 (0.32, 0.99)	0.057	0.68 (0.36, 1.19)	0.201
Other specialist	94 (1.6)	24 (1.3)	0.89 (0.55, 1.39)	0.629	0.89 (0.53, 1.44)	0.646
Unavailable	44 (0.8)	14 (0.8)	1.11 (0.58, 2.00)	0.732	1.53 (0.77, 2.88)	0.201
Number of patients per prescriber						
High (≥100 patients)	2306 (40.5)	783 (42.7)	—	—	—	—
Medium (10–100 patients)	1806 (31.7)	617 (33.6)	1.01 (0.89, 1.14)	0.921	0.94 (0.82, 1.08)	0.376
Low (<10 patients)	1585 (27.8)	435 (23.7)	0.81 (0.72, 0.92)	0.002	0.70 (0.59, 0.83)	<0.001
DAA regimen						
SOF/VEL	1771 (31.1)	653 (35.6)	—	—	—	—
SOF/LDV	1202 (21.1)	374 (20.4)	0.84 (0.73, 0.98)	0.023	0.94 (0.76, 1.16)	0.542
SOF+DCV	1213 (21.3)	434 (23.7)	0.97 (0.84, 1.12)	0.677	1.34 (1.07, 1.68)	0.010
PrOD	57 (1.0)	51 (2.8)	0.74 (0.42, 1.23)	0.264	0.65 (0.36, 1.14)	0.143
SOF+RBV	66 (1.2)	18 (1.0)	2.43 (1.64, 3.58)	<0.001	3.81 (2.18, 6.72)	<0.001
GRZ/ELB	379 (6.7)	97 (5.3)	0.54 (0.57, 0.88)	0.003	0.60 (0.45, 0.78)	<0.001
GLE/PIB	997 (17.5)	205 (11.2)	0.56 (0.47, 0.66)	<0.001	0.59 (0.45, 0.77)	<0.001
Other^a^	12 (0.2)	3 (0.2)	0.68 (0.15, 2.14)	0.548	0.82 (0.18, 2.81)	0.772
Ribavirin added to DAA regimen						
No	5566 (97.7)	1774 (96.7)	—	—		
Yes	131 (2.3)	61 (3.3)	1.46 (1.07, 1.98)	0.016	0.9 (0.57, 1.39)	0.642
Treatment duration						
8 wk	706 (12.4)	238 (13.0)	—	—	—	—
12 wk	3891 (68.3)	1411 (76.9)	1.08 (0.92, 1.26)	0.351	1.26 (0.98, 1.62)	0.067
16 or 24 wk	1100 (19.3)	186 (10.1)	0.50 (0.40, 0.62)	<0.001	0.82 (0.60, 1.12)	0.222
Treatment discontinuation status						
Late discontinuation	3494 (61.3)	596 (32.5)	—	—	—	—
Early discontinuation	2203 (38.7)	1239 (67.5)	3.29 (2.95, 3.69)	<0.001	3.91 (3.44, 4.44)	<0.001

^a^ Other includes glecaprevir/pibrentasvir+sofosbuvir; grazoprevir/elbasvir+sofosbuvir, paritaprevir/ritonavir/ombitasvir+dasabuvir+sofosbuvir, sofosbuvir/ledipasvir+daclatasvir.

^b^ OR calculated per 10-year decrease in age.

Abbreviations: DAA, direct-acting antiviral; GLE/PIB, glecaprevir/pibrentasvir; GRZ/ELB, grazoprevir/elbasvir; IQR, interquartile range; PrOD, paritaprevir/ritonavir/ombitasvir+dasabuvir; SOF+DCV, sofosbuvir+daclatasvir; SOF/LDV, sofosbuvir/ledipasvir; SOF+RBV, sofosbuvir+ribavirin±interferon; SOF/VEL; sofosbuvir/velpatasvir.

When analysis of factors associated with retreatment following treatment discontinuation was restricted to early discontinuation among those receiving GLE/PIB 8 weeks or SOF/VEL 12 weeks (n = 1762), those with early discontinuation of GLE/PIB 8 weeks had a lower likelihood of retreatment (adjusted HR = 0.62; 0.49, 0.79; *p* < 0.001; Table 4). Other factors were generally consistent with the original model. The pseudo-*R* ^2^ value for retreatment following treatment discontinuation model was 0.12.

**TABLE 4:** Factors associated with retreatment among people with early discontinuation of first-line pangenotypic regimens

	n (%)						
	Early discontinuation (n = 1762)	Not retreated (n = 1249)	Retreated (n = 513)	Unadjusted OR (95% CI)	*p*	Adjusted OR (95% CI)	*p*
Age [median (IQR)] (y)	42 (34, 51)	42 (34, 52)	43 (34, 50)	1.04 (0.96, 1.14)^a^	0.310	—	—
Sex							
Male	1084 (61.5)	737 (59.0)	347 (67.6)	—	—	—	—
Female	678 (38.5)	512 (41.0)	166 (32.4)	0.69 (0.55, 0.85)	0.001	0.67 (0.54, 0.84)	0.001
Concurrent treatment for HIV							
No	1733 (98.4)	1233 (98.7)	500 (97.5)	—	—	—	—
Yes	29 (1.6)	16 (1.3)	13 (2.5)	2.00 (0.94, 4.19)	0.065	1.86 (0.83, 4.10)	0.124
Location of patient residence							
Major city	1157 (65.7)	814 (65.2)	343 (66.9)	—	—		
Regional	537 (30.5)	390 (31.2)	147 (28.7)	0.89 (0.71, 1.21)	0.337		
Remote	42 (2.4)	28 (2.2)	14 (2.7)	1.87 (0.60, 2.24)	0.608		
Unavailable	26 (1.5)	17 (1.4)	9 (1.8)	1.26 (0.53, 2.78)	0.584		
Prescription payment type							
General	405 (23.0)	281 (22.5)	124 (24.2)	—	—		
Low-income subsidy	1357 (77.0)	968 (77.5)	389 (75.8)	0.91 (0.72, 1.16)	0.448		
Year treatment commenced							
2018	465 (26.4)	260 (20.8)	205 (40.0)	—	—	—	—
2019	510 (28.9)	350 (28.0)	160 (31.2)	0.58 (0.45, 0.95)	<0.001	0.66 (0.50, 0.87)	0.003
2020	364 (20.7)	272 (21.8)	92 (17.9)	0.43 (0.32, 0.58)	<0.001	0.51 (0.37, 0.70)	<0.001
2021	423 (24.0)	367 (29.4)	56 (10.9)	0.19 (0.14, 0.27)	<0.001	0.22 (0.15, 0.31)	<0.001
Prescriber type							
Gastroenterologist	331 (18.8)	225 (18.0)	106 (20.7)	—	—	—	—
Infectious diseases physician	94 (5.3)	65 (5.2)	29 (5.7)	0.95 (0.57, 1.54)	0.829	0.94 (0.55, 1.57)	0.809
General practitioner	1132 (64.2)	812 (65.0)	320 (62.4)	0.84 (0.64, 1.09)	0.186	1.03 (0.75, 1.42)	0.844
General physician	46 (2.6)	31 (2.5)	15 (2.9)	1.03 (0.52, 1.95)	0.937	1.00 (0.49, 1.96)	0.994
Sexual physician	32 (1.8)	23 (1.8)	9 (1.8)	0.83 (0.35, 1.80)	0.651	0.74 (0.30, 1.69)	0.490
Psychiatry or addiction specialist	30 (1.7)	18 (1.4)	12 (2.3)	1.42 (0.64, 3.02)	0.374	1.62 (0.70, 3.63)	0.248
Nurse practitioner	54 (3.1)	45 (3.6)	9 (1.8)	0.42 (0.19, 0.86)	0.026	0.62 (0.27, 1.31)	0.235
Other specialist	14 (0.8)	8 (0.6)	6 (1.2)	1.59 (0.51, 4.69)	0.400	1.93 (0.59, 6.11)	0.263
Unavailable	29 (1.6)	22 (1.8)	7 (1.4)	0.68 (0.26, 1.56)	0.382	1.18 (0.44, 2.85)	0.717
Number of patients per prescriber							
High (≥100 patients)	600 (34.1)	404 (32.3)	196 (38.2)	—	—	—	—
Medium (10–100 patients)	497 (28.2)	348 (27.9)	149 (29.0)	0.88 (0.68, 1.14)	0.340	0.91 (0.69, 1.21)	0.527
Low (<10 patients)	665 (37.7)	497 (39.8)	168 (32.7)	0.70 (0.54, 0.89)	0.004	0.81 (0.59, 1.10)	0.167
DAA regimen							
SOF/VEL 12 wk	1030 (58.5)	674 (54.0)	356 (69.4)	—	—	—	—
GLE/PIB 8 wk	732 (41.5)	575 (46.0)	157 (30.6)	0.52 (0.41, 0.64)	<0.001	0.62 (0.49, 0.79)	<0.001

^a^ OR calculated per 10-year decrease in age.

Abbreviations: DAA, direct-acting antiviral; GLE/PIB, glecepravir/pibrentasvir; IQR, interquartile range; SOF/VEL, sofosbuvir/velpatasvir.

### Retreatment discontinuation

A total of 7011 individuals commenced retreatment between 2016 and 2021 of whom 5231 were included in the assessment of retreatment discontinuation (Supplemental Figures 1 and 2; Supplemental Table 4, http://links.lww.com/HC9/A231). In the analysis population, the median age was 42 years (interquartile range: 33, 55) and most were male (81%). The most common DAA retreatment regimens were SOF/VEL (42%), GLE/PIB (27%), and sofosbuvir/velpatasvir/voxilaprevir (SOF/VEL/VOX; 18%).

Overall, 18% of those retreated discontinued retreatment, decreasing from 41% in the second half of 2016 to 14% in the second half of 2019 then increasing to 23% in the second half of 2021. Late discontinuation decreased from 24% in the second half of 2016 to 7% in 2019 and remained relatively stable (8% in the second half of 2021). Early discontinuation declined from 18% in the second half of 2016 to 6% in the second half of 2019, then increased to 15% in the second half of 2021. Of note, SOF/VEL/VOX became available in April 2019. In interrupted time series analysis, no impact of COVID-19 restrictions on retreatment discontinuation was observed (Supplemental Figure 4, http://links.lww.com/HC9/A231).

In adjusted Cox regression analysis, the factor associated with the greatest risk of retreatment discontinuation was initial treatment discontinuation (adjusted HR = 4.41; 3.85, 5.05; *p* < 0.001; Supplemental Table 5, http://links.lww.com/HC9/A231). Almost half (48%) of those who discontinued retreatment had also discontinued initial treatment. Retreatment with SOF/VEL/VOX (vs. SOF/VEL) was associated with decreased discontinuation risk (adjusted HR = 0.57; 95% CI: 0.43, 0.75; *p* < 0001). The *R* ^2^ value for the retreatment discontinuation model was 0.64.

## DISCUSSION

This analysis assessed DAA treatment and retreatment discontinuation using national pharmacy dispensing data in Australia. Treatment discontinuation more than doubled between 2016 and 2021, due to increases in early discontinuation. Early treatment discontinuation significantly increased the likelihood of retreatment, an indicator of treatment failure. Retreatment discontinuation rates halved during this period, following the availability of simplified pangenotypic and salvage DAA regimens to retreat virological failure, but remained higher than initial treatment discontinuation.

Increasing rates of treatment discontinuation may reduce the effectiveness of treatment as prevention-based HCV elimination efforts. The increasing trend in treatment discontinuation observed in this study corresponds with the expansion of treatment through diverse models of care and increasing treatment uptake among people who inject drugs.^6^,^12^ Between 2015 and 2021, the proportion of people who inject drugs attending needle and syringe programs reporting ever receiving HCV treatment increased from 11% to 62%.^12^ By 2021, 1 in 5 people aged under 35 (19%), an age group with higher rates of injecting drug use risk behaviors,^13^ discontinued treatment for HCV. Clinical trials and cohort studies report similar HCV treatment cure rates among people with prior or current injecting drug use regardless of model of care.^14^–^18^ Suboptimal adherence is often reported in these studies, although, treatment discontinuation appears uncommon. However, these studies are unlikely to be representative due to the impact of selection and observation biases. In addition, most are small in scale, with some reporting high losses to follow-up. Overlapping risk factors for treatment discontinuation and loss to follow-up are described^6^,^19^–^21^ and could lead to underestimations of treatment discontinuation in clinical studies. With all HCV treatment in Australia delivered through the PBS and half of the estimated population with HCV treated between 2016 and 2021,^22^ this analysis provides a highly representative picture of treatment discontinuation at a national level.

The use of simplified, short-duration therapies may reduce treatment discontinuation. Consistent with previous analyses assessing adherence, both longer treatment durations and DAA regimens with a higher number of daily pills increased the risk of treatment discontinuation.^18^,^23^–^25^ However, when analyses were restricted to first-line pangenotypic regimens SOF/VEL 12 weeks (1 pill daily) and GLE/PIB 8 weeks (3 pills daily), GLE/PIB 8 weeks was associated with lower discontinuation risk, suggesting duration of therapy is crucial. Although early discontinuation (at 4 wk) was less likely with longer durations, overall discontinuation risk increased markedly with longer durations of treatment, potentially reflecting waning levels of adherence over time even among those with more advanced disease states.

A quarter of those discontinuing initial treatment were retreated, with early discontinuation significantly increasing the likelihood of retreatment. Although the limitations of administrative data must be considered, a 38% reduction in the likelihood of retreatment among those with early discontinuation of GLE/PIB 8 weeks (vs. SOF/VEL 12 wk) may suggest lower treatment failure rates. Clinical trials have reported high efficacy of GLE/PIB 6 weeks for the treatment of recently acquired HCV infection, whereas trials assessing 6-week durations of SOF/VEL have reported suboptimal efficacy.^26^,^27^ The association of female sex with a lower likelihood of retreatment may be more complex. While it may reflect increased likelihood of viral clearance following discontinuation, it may also reflect lower levels of engagement with care among females treated for HCV.^28^,^29^

Initial treatment discontinuation was the strongest predictor of retreatment discontinuation. The inclusion of this variable in the retreatment discontinuation model resulted in significant improvements in the amount of variation in that was explained by the factors included in the model compared with the model for initial treatment discontinuation. It is estimated that 52% of those retreated in Australia between 2016 and 2021 were retreated for reinfection, and 48% for treatment failure.^4^ Retreatment with SOF/VEL/VOX, a simplified salvage regimen used to retreat virological failure was associated with decreased risk of retreatment discontinuation. However, those retreated with this regimen likely represent an older population with more advanced liver disease, whereas those retreated with first-line pangenotypic regimens likely represent a younger population with higher rates of initial treatment discontinuation and reinfection.^4^

No significant changes in the trends of treatment or retreatment discontinuation during the COVID-19 pandemic were identified. Less impacted by the COVID-19 pandemic than many other countries, Australian residents generally experienced minimal disruptions to health care services, with continued provision of in-clinic or telehealth consults. During this period, no changes in the trend of initial treatment uptake were observed,^30^ although modest decreases in national rates of HCV testing^31^ and the uptake of retreatment for reinfection^4^ were apparent during 2020.

There may be a broad range of intrapersonal, interpersonal, and structural factors that impact treatment discontinuation.^19^,^32^ Patient-centered interventions that promote adherence to treatment and retention in HCV care for at-risk populations^33^,^34^ will be critical if elimination is to be achieved. As the majority of prescribers in Australia, general practitioners play a critical role in HCV elimination efforts. Although a higher risk of discontinuation was observed for individuals treated by these prescribers, this may be more reflective of the populations accessing treatment through primary care. There are various resources available to support general practitioners treating HCV including DAA treatment guidelines, simplified HCV decision-making tools designed for primary care, live and on-demand training sessions, and programs of support and mentorship from HCV specialists.^9^,^35^–^38^ Additional resources surrounding adherence support and retreatment following discontinuation may be of benefit. In major cities care navigation through community-based, patient-centered services that provide comprehensive mental health, addiction, HCV treatment, and social support services is effective in providing adherence support to marginalized populations,^33^,^39^ however, access to such services in regional or remote areas is often limited.

Moving beyond resource-intensive support programs, the development of long-acting injectable DAAs, similar to those available for the treatment of HIV,^40^ may represent a viable alternative to oral-based treatments. In a recent study assessing preferences for HCV treatment among individuals with or at risk of HCV in 9 countries, 38% indicated a preference for long-acting injectable treatment over daily tablets taken orally.^41^ Despite consumer preference and clinical need for such treatment options the development of these treatments for HCV remains in its infancy.

This study had several strengths. The PBS data provides high-coverage pharmacy dispensing data at a national level and puts no burden on the populations being treated or health services to provide or collect study data. All individuals treated for HCV in Australia between 2016 and 2021 are captured in this dataset with exception of a small number of individuals who may have been treated through clinical trials or compassionate access programs for the PBS ineligible.^42^ The data are more representative than clinical trial or cohort study data that may be small in scale and impacted by numerous forms of bias. However, there are inherent limitations in the use of administrative data. Factors such as posttreatment HCV RNA testing, treatment outcome, drug and alcohol use, comorbid health conditions, homelessness, treatment side effects, and other factors that may predict discontinuation and the uptake of retreatment were not available in this dataset.^19^,^33^,^43^ While pharmaceutical dispensing data has been shown to be a reliable measure of adherence in other studies^44^,^45^ dispensation occurred in 28-day intervals and it is possible that those with treatment dispensed did not commence treatment or had suboptimal adherence. It is also possible there was some impact of misclassification bias due to clinician-directed shortening of longer duration courses due to early response. During the study period an increasing proportion of individuals received their entire treatment (7%) or retreatment (25%) course in a single dispensation and were excluded from this analysis. As factors associated with treatment discontinuation were more prevalent among those who dispensed their entire treatment course at once, the rates of discontinuation reported in this analysis may be an underestimation.

Increasing rates of treatment discontinuation have the potential to negatively impact HCV elimination efforts. While many of those discontinuing treatment have likely achieved cure, there may be a significant proportion of people who have failed treatment, disengaged from care, and remain viraemic. Access to adherence support and retreatment will be critical for those with initial treatment discontinuation.

## Supplementary Material
