# Supplementary material for: Increasing national trend of direct-acting antiviral discontinuation among people treated for HCV 2016–2021
Source: Hepatol Commun. 2023 Mar 30;7(4):e0125. doi: 10.1097/HC9.0000000000000125 (PMC10069828; doi:10.1097/HC9.0000000000000125)
Supplement: Supplementary file 1 [file hc9-7-e0125-s001.docx]

**Increasing national trend of direct acting antiviral discontinuation among people treated for hepatitis C virus 2016-2021**

Joanne Carson1, Sebastiano Barbieri2, Gail V Matthews1, Gregory J Dore1, Behzad Hajarizadeh1

1 The Kirby Institute, UNSW Sydney, Sydney, Australia
2 Centre for Big Data Research in Health, UNSW Sydney, Sydney, Australia

**Table of contents**

| Section | Page |
| --- | --- |
| DAA treatment in Australia | 2 |
| DAA treatment and retreatment uptake | 3 |
| DAA prescription dispensation patterns | 4 |
| Characteristics of individuals excluded from the treatment discontinuation analysis | 5 |
| Prescriber caseloads | 7 |
| Factors associated with early treatment discontinuation | 8 |
| The impact of COVID-19 restrictions on treatment and retreatment discontinuation | 10 |
| Characteristics of individuals excluded from the retreatment discontinuation analysis | 11 |
| Factors associated with retreatment discontinuation | 13 |
| Supplementary references | 14 |

**DAA TREATMENT IN AUSTRALIA**

In Australia, there has been unrestricted access to government subsidized direct acting antiviral therapies (DAAs) since 2016, including prescription by any medical practitioner, and no restrictions by ongoing drug use or number of times an individual can be retreated (1,2). All DAA treatment and retreatment dispensation is reported through the Australian Pharmaceutical Benefit Scheme (PBS). The PBS provides high-coverage, structured information on patient demographics and pharmacy dispensing. DAA treatment is typically dispensed in 28-dose packages (4-weeks supply at a time). The authorised duration of treatment, initial supply and the filling of repeat prescriptions is reported through PBS allowing for assessment of discontinuation.

**Supplementary Table 1.** DAA regimens and PBS listing dates

| DAA regimen  (±ribavirin unless stated) | Abbreviation | Category | Date listed | Authorised durations | Number of pills daily |
| --- | --- | --- | --- | --- | --- |
| sofosbuvir/ledipasvir | SOF/LDV | Genotype specific | 1-Mar-16 | 8, 12, 24 weeks | 1 |
| sofosbuvir+daclatasvir | SOF+DCV | Genotype specific | 1-Mar-16 | 12, 24 weeks | 2 |
| sofosbuvir+ribavirin | SOF+RBV | Genotype specific | 1-Mar-16 | 12, 24 weeks | 2 |
| sofosbuvir+interferon+ribavirin | SOF+IFN+RBV | Genotype specific | 1-Mar-16 | 12, 24 weeks | 3 |
| paritaprevir/ritonavir/ombitasvir +dasabuvir | PrOD | Genotype specific | 1-May-16 | 12, 24 weeks | 4 |
| grazoprevir/elbasvir | GRZ/ELB | Genotype specific | 1-Jan-17 | 12, 16 weeks | 1 |
| sofosbuvir/velpatasvir | SOF/VEL | Pangenotypic | 1-Aug-17 | 12 weeks | 3 |
| glecaprevir/pibrentasvir | GLE/PIB | Pangenotypic/  Salvage^ | 1-Aug-18 | 8, 12, 16 weeks | 1 |
| sofosbuvir/velpatasvir/voxilaprevir | SOF/VEL/VOX | Salvage | 1-Apr-19 | 12 weeks | 1 |
| grazoprevir/elbasvir+sofosbuvir | GRZ/ELB+SOF | Salvage | NA | NA | 2 |
| glecaprevir/pibrentasvir+sofosbuvir | GLE/PIB+SOF | Salvage | NA | NA | 4 |
| paritaprevir/ritonavir/ombitasvir +dasabuvir+sofosbuvir | PrOD+SOF | Salvage | NA | NA | 5 |
| sofosbuvir/ledipasvir+daclatasvir | SOF/LDV+DCV | Salvage | NA | NA | 2 |

A total of 95,275 individuals commenced treatment between 2016-2021 and 7,011 individuals commenced retreatment. Treatment uptake declined from 32,274 in 2016 to 5,349 in 2021 (**Supplementary Figure 1A**). Retreatment uptake increased from 80 in 2016 to 1530 in 2019 then declined to 1025 in 2021 **(Supplementary Figure 1B).**

**DAA TREATMENT AND RETREATMENT UPTAKE**

**Supplementary Figure 1.** DAA treatment and retreatment uptake in Australia 2016-2021 plotted in six monthly intervals (A) treatment uptake, (B) retreatment uptake

**
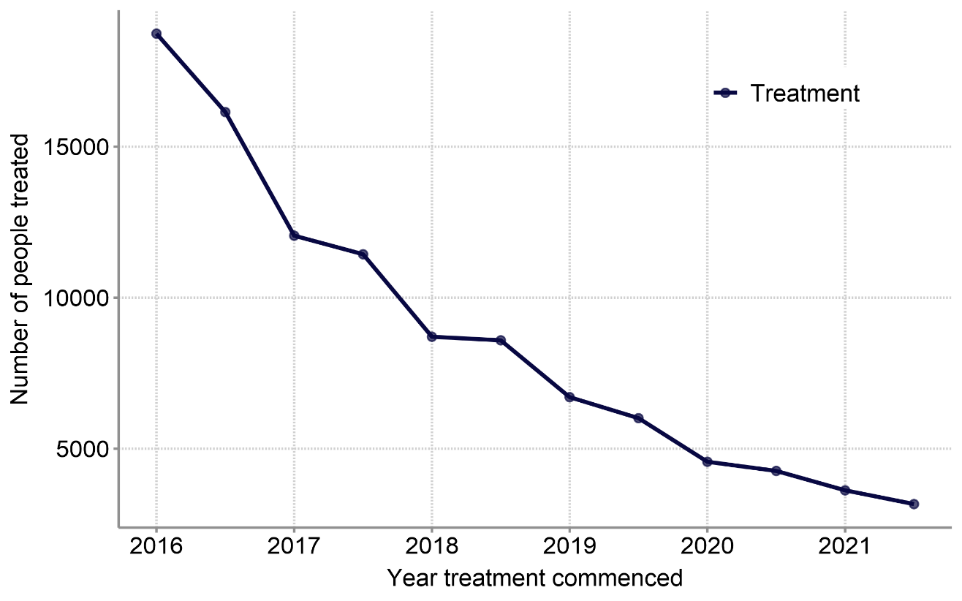
**

**(A)**


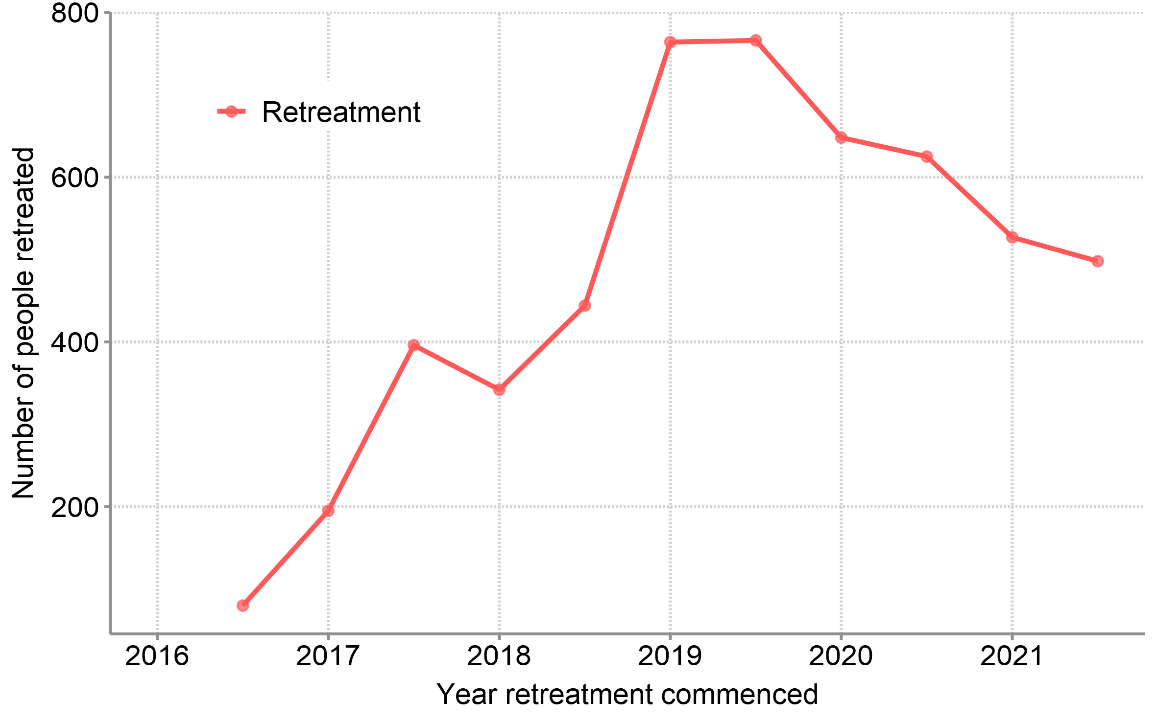
**(B)**

**PRESCRIPTION DISPENSATION PATTERNS**

The majority of individuals received a full standard treatment course (28-day dosage dispensed multiple times until authorised duration was complete). A small proportion of people received extended duration of treatment (multiple scripts totalling >4 weeks than maximum authorised duration for the specific DAA regimen) or received multiple prescriptions for a standard DAA course (indicative of a lost prescription or treatment switch). There has been increasing single dispensation of a full DAA courses over time (all 28-day prescription repeats dispensed at the same time). For treatment, single dispensation of entire treatment courses occurred for 7% (n=6289), increasing from <1% in the first half of 2016 to 17% in the second half of 2021 (**Supplementary Figure 2A**). For retreatment, single dispensation of the entire retreatment course occurred for 21% (n=1503), increasing from 1% in the second half of 2016 to 34% in the second half of 2021 (**Supplementary Figure 2B**). Treatment and retreatment discontinuation could not be assessed among individuals with a single dispensation of the full DAA course and these individuals were excluded from analyses assessing discontinuation.

**Supplementary Figure 2.** Prescription dispensation status (A) Treatment dispensation status, (B) Retreatment dispensation status plotted at 6-monthly intervals


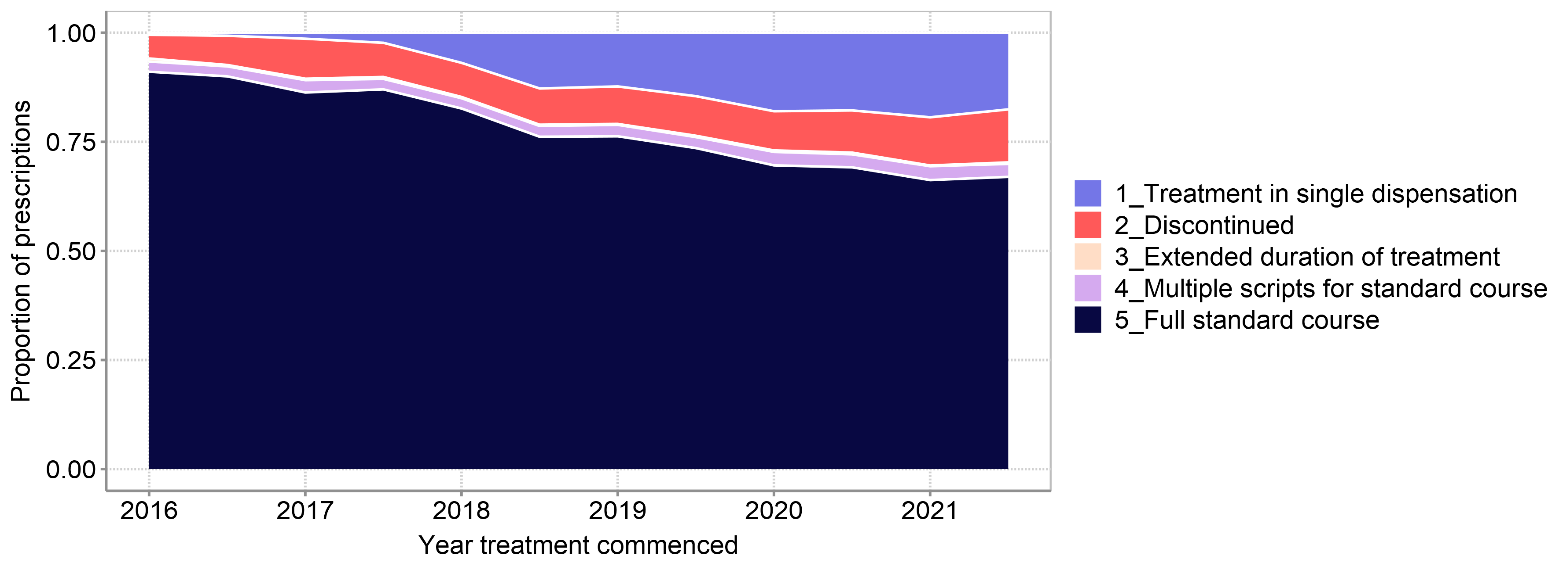


**(A)**

**
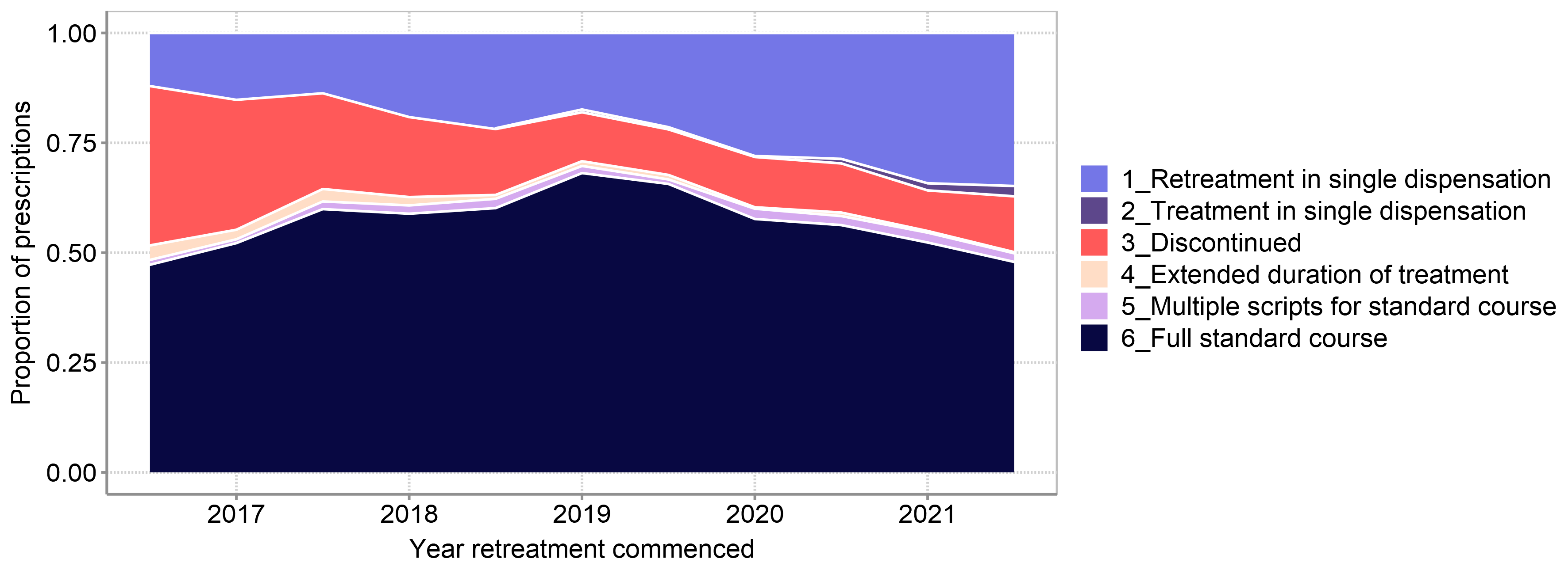
**

**(B)**

**CHARACTERISTICS OF INDIVIDUALS EXCLUDED FROM THE DISCONTINUATION ANALYSES**

**Supplementary Table 2.** Characteristics of individuals with a full standard treatment course dispensed, treatment discontinuation and entire treatment course dispensed in a single dispensation

|  | Treatment fully  dispensed*  (n=81454) | Treatment  discontinued  (n=7532) | Single dispensation  of full course^$^ (n=6289) | Total treated population (n=95275) |
| --- | --- | --- | --- | --- |
| Age in years, (median [IQR]) | 49 [40, 57] | 45 [36, 54] | 35 [28, 43] | 48 [39, 57] |
| Female sex, n (%) | 26563 (32.6) | 2571 (34.1) | 995 (15.8) | 30129 (31.6) |
| Concurrent treatment for HIV, n (%) | 1411 ( 1.7) | 133 ( 1.8) | 50 ( 0.8) | 1594 ( 1.7) |
| Location of patient residence |  |  |  |  |
| Major city | 52665 (64.7) | 4818 (64.0) | 3374 (53.6) | 60857 (63.9) |
| Regional | 26189 (32.2) | 2458 (32.6) | 2489 (39.6) | 31136 (32.7) |
| Remote | 1205 ( 1.5) | 147 ( 2.0) | 148 ( 2.4) | 1500 ( 1.6) |
| Unavailable | 1395 ( 1.7) | 109 ( 1.4) | 278 ( 4.4) | 1782 ( 1.9) |
| Low-income subsidy prescription, n (%) | 48749 (59.8) | 5320 (70.6) | 702 (11.2) | 54771 (57.5) |
| Year treatment commenced, n (%) |  |  |  |  |
| 2016 | 30321 (37.2) | 1953 (25.9) | 183 ( 2.9) | 32457 (34.1) |
| 2017 | 19052 (23.4) | 1809 (24.0) | 388 ( 6.2) | 21249 (22.3) |
| 2018 | 12614 (15.5) | 1237 (16.4) | 1504 (23.9) | 15355 (16.1) |
| 2019 | 8897 (10.9) | 1008 (13.4) | 1528 (24.3) | 11433 (12.0) |
| 2020 | 5980 ( 7.3) | 766 (10.2) | 1470 (23.4) | 8216 ( 8.6) |
| 2021 | 4590 ( 5.6) | 759 (10.1) | 1216 (19.3) | 6565 ( 6.9) |
| Prescriber type, n (%) |  |  |  |  |
| Gastroenterologist | 30373 (37.3) | 2082 (27.6) | 1505 (23.9) | 33960 (35.6) |
| Infectious Diseases Physician | 5037 ( 6.2) | 440 ( 5.8) | 633 (10.1) | 6110 ( 6.4) |
| General Practitioner | 35663 (43.8) | 4089 (54.3) | 3087 (49.1) | 42839 (45.0) |
| General Physician | 5614 ( 6.9) | 408 ( 5.4) | 89 ( 1.4) | 6111 ( 6.4) |
| Sexual Physician | 1117 ( 1.4) | 125 ( 1.7) | 35 ( 0.6) | 1277 ( 1.3) |
| Psychiatry or Addiction specialist | 920 ( 1.1) | 107 ( 1.4) | 10 ( 0.2) | 1037 ( 1.1) |
| Nurse Practitioner | 873 ( 1.1) | 105 ( 1.4) | 508 ( 8.1) | 1486 ( 1.6) |
| Other Specialist | 1164 ( 1.4) | 118 ( 1.6) | 264 ( 4.2) | 1546 ( 1.6) |
| Unavailable | 693 ( 0.9) | 58 ( 0.8) | 158 ( 2.5) | 909 ( 1.0) |
| HCV prescriber experience, n (%) |  |  |  |  |
| High (≥100 patients) | 38494 (47.3) | 3089 (41.0) | 5410 (86.0) | 46993 (49.3) |
| Medium (10-100 patients) | 26636 (32.7) | 2423 (32.2) | 663 (10.5) | 29722 (31.2) |
| Low (<10 patients) | 16324 (20.0) | 2020 (26.8) | 216 ( 3.4) | 18560 (19.5) |
| DAA regimen, n (%) |  |  |  |  |
| SOF/VEL | 23794 (29.2) | 2424 (32.2) | 2538 (40.4) | 28756 (30.2) |
| SOF/LDV | 25470 (31.3) | 1576 (20.9) | 562 ( 8.9) | 27608 (29.0) |
| SOF+DCV | 17039 (20.9) | 1647 (21.9) | 145 ( 2.3) | 18831 (19.8) |
| SOF+RBV | 1775 ( 2.2) | 108 ( 1.4) | 6 ( 0.1) | 1889 ( 2.0) |
| PrOD | 504 ( 0.6) | 84 ( 1.1) | 0 ( 0.0) | 588 ( 0.6) |
| GRZ/ELB | 3714 ( 4.6) | 476 ( 6.3) | 81 ( 1.3) | 4271 ( 4.5) |
| GLE/PIB | 9069 (11.1) | 1202 (16.0) | 2950 (46.9) | 13221 (13.9) |
| Other^ | 89 ( 0.1) | 15 ( 0.2) | 7 ( 0.1) | 111 ( 0.1) |
| Ribavirin added to DAA regimen, n (%) | 2503 ( 3.1) | 192 ( 2.5) | 9 ( 0.1) | 2704 ( 2.8) |
| Treatment duration, n (%) |  |  |  |  |
| 8-weeks | 12854 (15.8) | 944 (12.5) | 3187 (50.7) | 16985 (17.8) |
| 12-weeks | 61414 (75.4) | 5302 (70.4) | 3070 (48.8) | 69786 (73.2) |
| 16-weeks | 155 ( 0.2) | 167 ( 2.2) | 7 ( 0.1) | 329 ( 0.3) |
| 24-weeks | 7031 ( 8.6) | 1119 (14.9) | 25 ( 0.4) | 8175 ( 8.6) |
| Received retreatment, n (%) | 4695 ( 5.8) | 1835 (24.4) | 1137 (18.1) | 7667 ( 8.0) |

**Abbreviations:** DAA, direct acting antiviral; SOF/VEL; sofosbuvir/velpatasvir; SOF/LDV, sofosbuvir/ledipasvir; SOF+DCV, sofosbuvir+daclatasvir; SOF±IFN, sofosbuvir±interferon; PrOD, paritaprevir/ritonavir/ombitasvir+dasabuvir; GRZ/ELB, grazoprevir/elbasvir; GLE/PIB, glecaprevir/pibrentasvir;

* Treatment fully dispensed includes individuals receiving a full standard treatment course, extended duration of treatment and those receiving multiple prescriptions for a standard treatment course.

^$^ Single dispensation of a single treatment course includes individuals receiving a full course of treatment or retreatment in a single dispensation. These individuals were excluded from the retreatment discontinuation analysis.

^Other includes glecaprevir/pibrentasvir+sofosbuvir; grazoprevir/elbasvir+sofosbuvir, paritaprevir/ritonavir/ombitasvir+dasabuvir+sofosbuvir, sofosbuvir/ledipasvir+daclatasvir

Compared to those with a standard course of treatment (multiple 28-day repeats) and those that discontinued treatment, those with a with a single dispensation of a full treatment course were younger (49 vs. 45 vs. 35 years), a higher proportion were residing in regional locations (32% vs. 33% vs. 40%) and a lower proportion were receiving low-income subsidy prescriptions (60% vs. 71% vs. 11%).

Compared to those with a standard course of treatment and those that discontinued treatment, a higher proportion of those with a single dispensation of a full treatment course were treated by nurse practitioners (1% vs 1% vs 8%) and highly experienced HCV prescribers (47% vs 41% vs 86%). A higher proportion received SOF/VEL (29% vs 32% vs 40%) or GLE/PIB (11% vs 16% vs 47%) and 8-week treatment durations (16% vs 13% vs 51%).

The proportion of retreatment among those with a single dispensation of a full treatment course (18%) was higher than those with a standard course dispensed (6%) and lower than those that discontinued treatment (24%).

As the incarcerated population is predominantly comprised of younger males and that incarcerated individuals are ineligible for low-income subsidies (the cost of DAA medications is covered by the prison health service) (3) it is likely that the bulk of single dispensations of full treatment courses are occurring in this setting. There is a high prevalence and incidence of HCV within Australian prisons (4). High caseload HCV prescribers may visit prisons to provide health services. Additionally, nurse led treatment programs have expanded significantly within the Australian prison system (5). Treatment was scaled up later within prisons compared to community settings and is predominantly with first line pangenotypics GLE/PIB 8-weeks or SOF/VEL 12-weeks.

Given that many of the factors associated with treatment discontinuation were prevalent among those with a single dispensation of treatment and that there are frequent movements between prisons and community settings it is possible this population has increased risk of treatment discontinuation.

**PRESCRIBER CASELOADS**

The relationship between duration of prescribing and the number of prescriptions written by each prescriber is displayed in **Supplementary Figure 3**. Between March 2016 to December 2021, there were 10,543 unique prescribers of DAA therapy in Australia. Most prescribers were general practitioners (87%), followed by HCV specialists (8%) and other specialists (5%).

Overall, the median number of patients treated was 2 (IQR; 1, 4; range 1, 1,572) and the median time from first DAA prescription was 4.0 years (IQR 2.4, 5.1; range 1 day to 5.8 years).

HCV specialists treated a higher median number of patients (9 [IQR 2, 51]) than general practitioners (1 [IQR 1,1]) and other specialists (2 [IQR 1,12]). The median time from first DAA prescription was higher among HCV specialists (5.5 years [IQR 3.2, 5.8]) compared to general practitioners (3.9 years [IQR 2.4, 4.9]) and other specialists (4.1 years [IQR 2.1, 5.6]).

While a modest correlation between time prescribing and number of patients treated is observable, the majority of prescribers remained in the low caseload (<10 patients treated) category irrespective of time since the first DAA prescription was written.

**
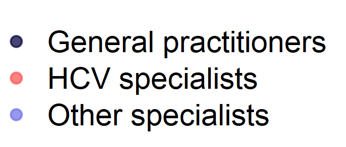

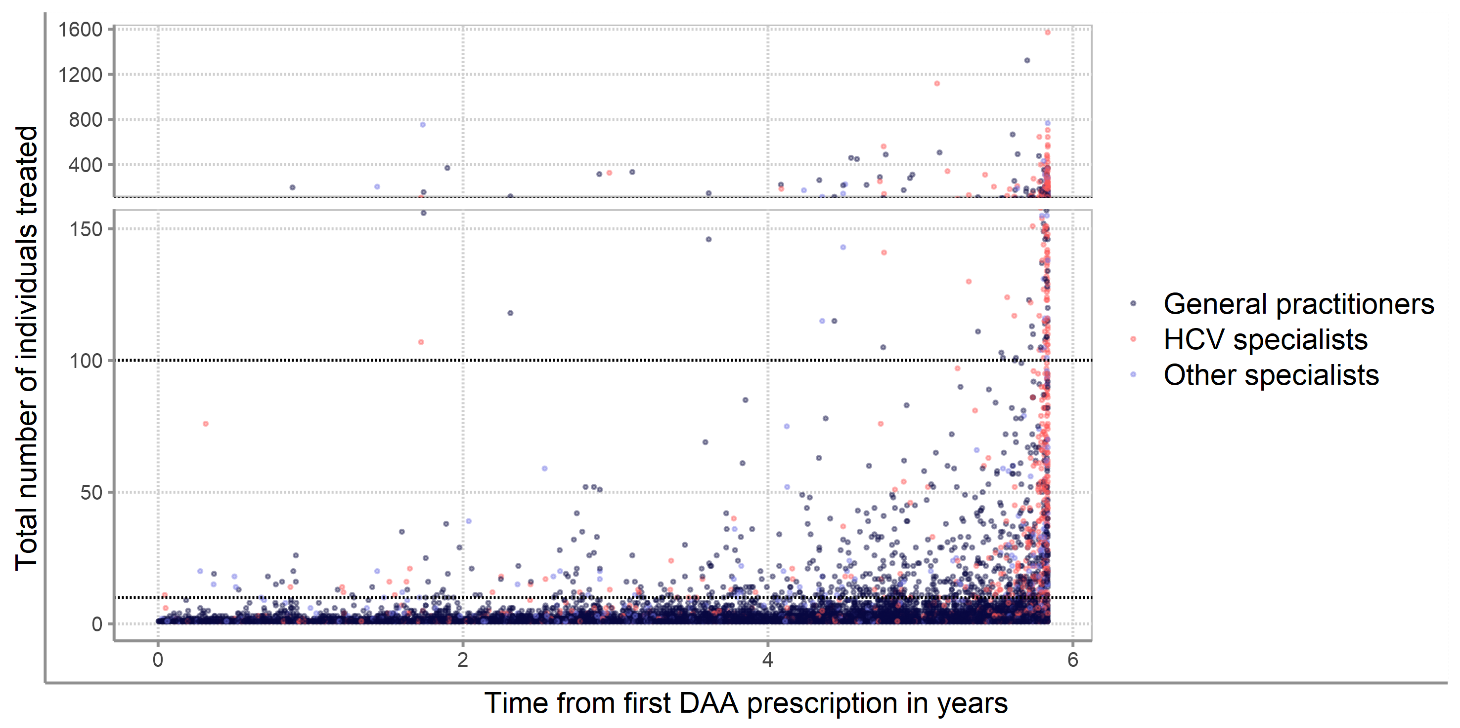
**

**Supplementary Figure 3.** Time from first DAA prescription by the number of individuals treated

**Abbreviations:** HCV, hepatitis C virus

HCV specialists included gastroenterologists and infectious diseases physicians. Other specialists included general physicians, sexual health physicians, psychiatry or addiction specialists, nurse practitioners and other or unknown.

**Supplementary Table 3.** Factors associated with early treatment discontinuation

|  | No early  discontinuation (n=85,545) | Early  discontinuation (n=3,441) | Unadjusted odds ratio, 95%CI | p value | Adjusted odds ratio, 95%CI | p value |
| --- | --- | --- | --- | --- | --- | --- |
| Age in years (median [IQR]) | 49 [40, 57] | 44 [35, 53] | 1.36 (1.32, 1.40)* | <0.001 | 1.27 (1.24, 1.31)* | <0.001 |
| Sex |  |  |  |  |  |  |
| Male | 57655 (67.4) | 2197 (63.8) | - | - | - | - |
| Female | 27890 (32.6) | 1244 (36.2) | 1.17 (1.09, 1.26) | <0.001 | 1.11 (1.03, 1.19) | 0.005 |
| Concurrent treatment for HIV |  |  |  |  |  |  |
| No | 84058 (98.3) | 3384 (98.3) | - | - |  |  |
| Yes | 1487 ( 1.7) | 57 ( 1.7) | 0.95 (0.72, 1.23) | 0.719 |  |  |
| Location of patient residence |  |  |  |  |  |  |
| Major city | 55233 (64.6) | 2251 (65.4) | - | - | - | - |
| Regional | 27576 (32.2) | 1070 (31.1) | 0.95 (0.88, 1.03) | 0.194 | 0.88 (0.81, 0.95) | 0.001 |
| Remote | 1276 ( 1.5) | 76 ( 2.2) | 1.46 (1.15, 1.83) | 0.002 | 1.38 (1.08, 1.75) | 0.008 |
| Unavailable | 1460 ( 1.7) | 44 ( 1.3) | 0.74 (0.54, 0.99) | 0.051 | 0.63 (0.46, 0.85) | 0.003 |
| Prescription payment type |  |  |  |  |  |  |
| General | 34031 (39.8) | 884 (25.7) | - | - | - | - |
| Low-income subsidy | 51514 (60.2) | 2557 (74.3) | 1.91 (1.77, 2.07) | <0.001 | 1.87 (1.72, 2.02) | <0.001 |
| Year treatment commenced, n (%) |  |  |  |  |  |  |
| 2016 | 31718 (37.1) | 556 (16.2) | - | - | - | - |
| 2017 | 20093 (23.5) | 768 (22.3) | 2.18 (1.95, 2.44) | <0.001 | 1.78 (1.57, 2.01) | <0.001 |
| 2018 | 13201 (15.4) | 650 (18.9) | 2.81 (2.50, 3.15) | <0.001 | 2.22 (1.90, 2.60) | <0.001 |
| 2019 | 9309 (10.9) | 596 (17.3) | 3.65 (3.25, 4.11) | <0.001 | 2.69 (2.26, 3.19) | <0.001 |
| 2020 | 6334 ( 7.4) | 412 (12.0) | 3.71 (3.26, 4.23) | <0.001 | 2.65 (2.20, 3.18) | 0.000 |
| 2021 | 4890 ( 5.7) | 459 (13.3) | 5.35 (4.71, 6.08) | <0.001 | 3.87 (3.21, 4.66) | <0.001 |
| Prescriber type |  |  |  |  |  |  |
| Gastroenterologist | 31643 (37.0) | 812 (23.6) | - | - | - | - |
| Infectious Diseases Physician | 5300 ( 6.2) | 177 ( 5.1) | 1.30 (1.10, 1.53) | 0.002 | 1.10 (0.93, 1.30) | 0.268 |
| General Practitioner | 37679 (44.0) | 2073 (60.2) | 2.14 (1.98, 2.33) | <0.001 | 1.41 (1.28, 1.56) | <0.001 |
| General Physician | 5887 ( 6.9) | 135 ( 3.9) | 0.89 (0.74, 1.07) | 0.232 | 0.95 (0.78, 1.14) | 0.585 |
| Sexual Health Physician | 1184 ( 1.4) | 58 ( 1.7) | 1.91 (1.44, 2.48) | <0.001 | 1.45 (1.09, 1.90) | 0.008 |
| Psychiatry or addiction specialist | 972 ( 1.1) | 55 ( 1.6) | 2.21 (1.65, 2.89) | <0.001 | 1.20 (0.89, 1.59) | 0.204 |
| Nurse Practitioner | 921 ( 1.1) | 57 ( 1.7) | 2.41 (1.81, 3.15) | <0.001 | 0.97 (0.73, 1.28) | 0.858 |
| Other Specialist | 1242 ( 1.5) | 40 ( 1.2) | 1.26 (0.90, 1.71) | 0.167 | 0.90 (0.64, 1.23) | 0.522 |
| Unavailable | 717 ( 0.8) | 34 ( 1.0) | 1.85 (1.28, 2.58) | 0.001 | 0.81 (0.56, 1.14) | 0.248 |
| Number of patients per prescriber |  |  |  |  |  |  |
| High (≥100 patients) | 40271 (47.1) | 1312 (38.1) | - | - | - | - |
| Medium (10-100 patients) | 27982 (32.7) | 1077 (31.3) | 1.18 (1.09, 1.28) | <0.001 | 1.00 (0.91, 1.09) | 0.953 |
| Low (<10 patients) | 17292 (20.2) | 1052 (30.6) | 1.87 (1.72, 2.03) | <0.001 | 1.06 (0.96, 1.17) | 0.276 |
| DAA regimen |  |  |  |  |  |  |
| SOF/VEL | 25029 (29.3) | 1189 (34.6) | - | - | - | - |
| SOF/LDV | 26422 (30.9) | 624 (18.1) | 0.50 (0.45, 0.54) | <0.001 | 0.97 (0.84, 1.12) | 0.722 |
| SOF+DCV | 18212 (21.3) | 474 (13.8) | 0.55 (0.49, 0.61) | <0.001 | 1.10 (0.94, 1.27) | 0.242 |
| PrOD | 1842 ( 2.2) | 41 ( 1.2) | 1.74 (1.26, 2.35) | <0.001 | 4.55 (3.23, 6.27) | <0.001 |
| SOF±IFN | 543 ( 0.6) | 45 ( 1.3) | 0.47 (0.34, 0.63) | <0.001 | 1.28 (0.83, 1.95) | 0.257 |
| GRZ/ELB | 3961 ( 4.6) | 229 ( 6.7) | 1.22 (1.05, 1.40) | 0.008 | 1.48 (1.26, 1.73) | <0.001 |
| GLE/PIB | 9439 (11.0) | 832 (24.2) | 1.86 (1.69, 2.03) | <0.001 | 1.16 (0.99, 1.37) | 0.073 |
| Other | 97 ( 0.1) | 7 ( 0.2) | 1.52 (0.64, 3.05) | 0.287 | 1.95 (0.81, 3.96) | 0.092 |
| Ribavirin added to DAA regimen |  |  |  |  |  |  |
| No | 82910 (96.9) | 3381 (98.3) | - | - | - | - |
| Yes | 2635 ( 3.1) | 60 ( 1.7) | 0.56 (0.43, 0.72) | <0.001 | 0.89 (0.62, 1.25) | 0.522 |
| Treatment duration |  |  |  |  |  |  |
| 8-weeks | 12854 (15.0) | 944 (27.4) | - | - | - | - |
| 12-weeks | 64385 (75.3) | 2331 (67.7) | 0.49 (0.46, 0.53) | <0.001 | 0.72 (0.63, 0.84) | <0.001 |
| 16 or 24-weeks | 8306 ( 9.7) | 166 ( 4.8) | 0.27 (0.23, 0.32) | <0.001 | 0.74 (0.59, 0.92) | 0.007 |

**Abbreviations:** DAA, direct acting antiviral; SOF/VEL; sofosbuvir/velpatasvir; SOF/LDV, sofosbuvir/ledipasvir; SOF+DCV, sofosbuvir+daclatasvir; SOF+RBV, sofosbuvir+ribavirin±interferon; PrOD, paritaprevir/ritonavir/ombitasvir+dasabuvir; GRZ/ELB, grazoprevir/elbasvir; GLE/PIB, glecaprevir/pibrentasvir;

^Other includes glecaprevir/pibrentasvir+sofosbuvir; grazoprevir/elbasvir+sofosbuvir, paritaprevir/ritonavir/ombitasvir+dasabuvir+sofosbuvir, sofosbuvir/ledipasvir+daclatasvir

* Odds ratio calculated per ten-year decrease in age

**THE IMPACT OF COVID-19 RESTRICTIONS ON TREATMENT AND RETREATMENT DISCONTINUATION**

Interrupted time series regression analysis was used to assess the impact of COVID-19 restrictions on (re)treatment discontinuation with adjustment for calendar time (1-monthly intervals)(9). COVID-19 pandemic restrictions were considered as occurring between March 2020 to September 2021, although restrictions occurred intermittently in Australian jurisdictions during this period. In interrupted time series regression analysis, no impact of COVID-19 restrictions were observed for treatment (p=0.2272) or retreatment discontinuation (p=0.5695)

**Supplementary figure 4. Trends in treatment and retreatment discontinuations** (A) trends in treatment discontinuation (B) trends in retreatment discontinuation assessed by calendar month in interrupted time series analysis.


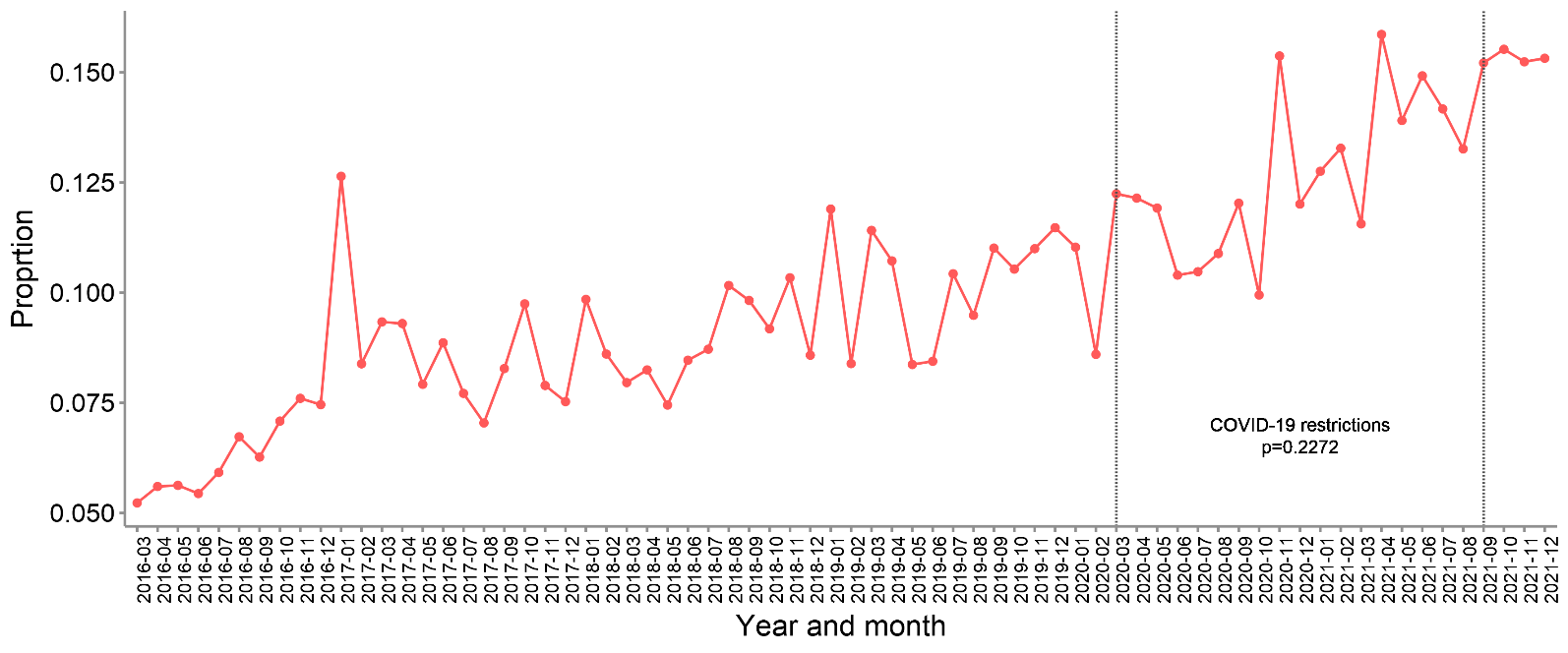
**(A)**


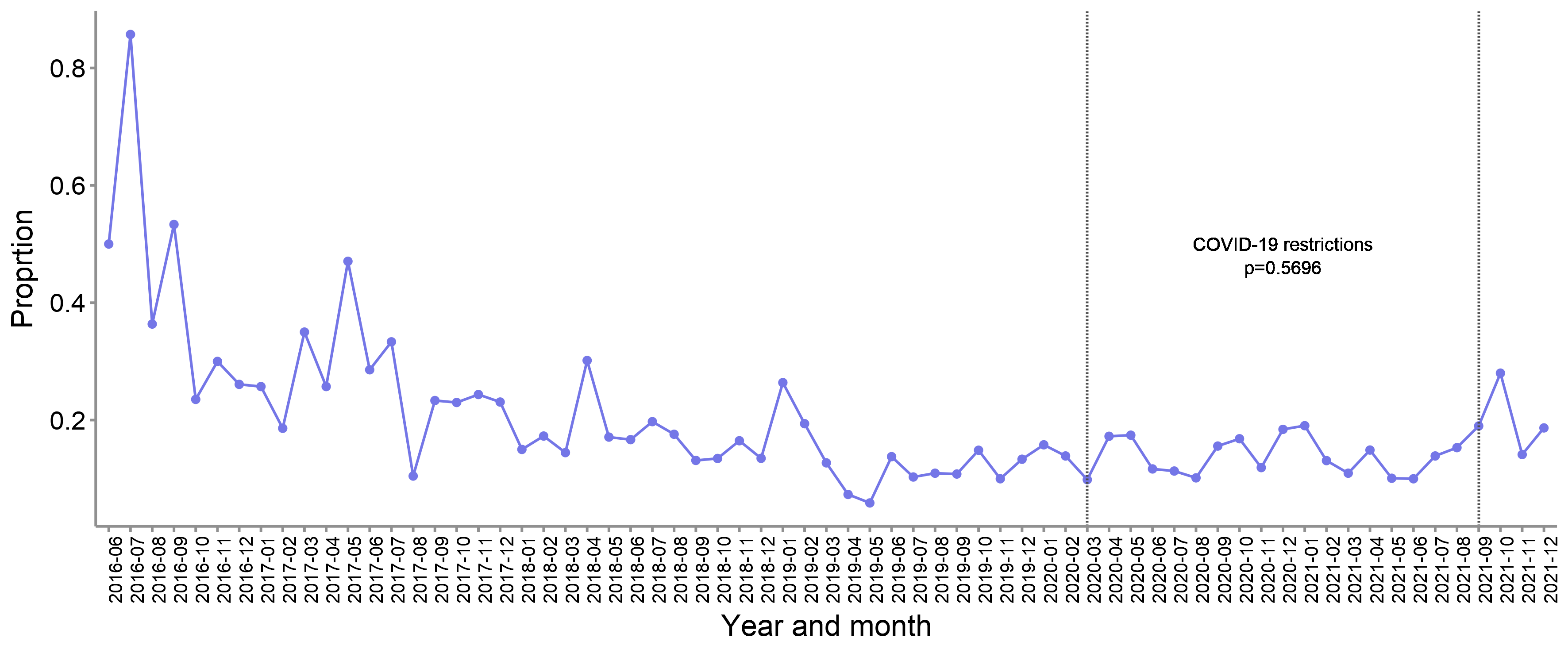
**(B)**

**Supplementary Table 4.** Characteristics of individuals with a full standard retreatment course dispensed, retreatment discontinuation and entire retreatment course dispensed in a single dispensation

|  | Retreatment fully  dispensed*  (n=4292) | Retreatment  discontinued  (n=939) | Single dispensation  of full course^$^ (n=1780) | Total retreated population (n=7011) |
| --- | --- | --- | --- | --- |
| Age in years, (median [IQR]) | 42 [32, 52] | 43 [35, 52] | 35 [28, 45] | 40 [31, 50] |
| Female sex, n (%) | 714 (16.6) | 271 (28.9) | 328 (18.4) | 1313 (18.7) |
| Concurrent treatment for HIV, n (%) | 98 ( 2.3) | 22 ( 2.3) | 23 ( 1.3) | 143 ( 2.0) |
| Location of patient residence |  |  |  |  |
| Major city | 2580 (60.1) | 620 (66.0) | 1060 (59.6) | 4260 (60.8) |
| Regional | 1504 (35.0) | 282 (30.0) | 629 (35.3) | 2415 (34.4) |
| Remote | 88 ( 2.1) | 20 ( 2.1) | 46 ( 2.6) | 154 ( 2.2) |
| Unavailable | 120 ( 2.8) | 17 ( 1.8) | 45 ( 2.5) | 182 ( 2.6) |
| Low-income subsidy prescription, n (%) | 2131 (49.7) | 633 (67.4) | 657 (36.9) | 3421 (48.8) |
| Year treatment commenced, n (%) |  |  |  |  |
| 2016 | 47 ( 1.1) | 33 ( 3.5) | 11 ( 0.6) | 91 ( 1.3) |
| 2017 | 423 ( 9.9) | 168 (17.9) | 98 ( 5.5) | 689 ( 9.8) |
| 2018 | 623 (14.5) | 162 (17.3) | 205 (11.5) | 990 (14.1) |
| 2019 | 1315 (30.6) | 204 (21.7) | 381 (21.4) | 1900 (27.1) |
| 2020 | 1061 (24.7) | 201 (21.4) | 514 (28.9) | 1776 (25.3) |
| 2021 | 823 (19.2) | 171 (18.2) | 571 (32.1) | 1565 (22.3) |
| Prescriber type, n (%) |  |  |  |  |
| Gastroenterologist | 1351 (31.5) | 217 (23.1) | 371 (20.8) | 1939 (27.7) |
| Infectious Diseases Physician | 413 ( 9.6) | 55 ( 5.9) | 147 ( 8.3) | 615 ( 8.8) |
| General Practitioner | 1885 (43.9) | 566 (60.3) | 893 (50.2) | 3344 (47.7) |
| General Physician | 169 ( 3.9) | 26 ( 2.8) | 22 ( 1.2) | 217 ( 3.1) |
| Sexual Physician | 62 ( 1.4) | 9 ( 1.0) | 21 ( 1.2) | 92 ( 1.3) |
| Psychiatry or Addiction specialist | 57 ( 1.3) | 20 ( 2.1) | 25 ( 1.4) | 102 ( 1.5) |
| Nurse Practitioner | 164 ( 3.8) | 24 ( 2.6) | 219 (12.3) | 407 ( 5.8) |
| Other Specialist | 64 ( 1.5) | 7 ( 0.7) | 33 ( 1.9) | 104 ( 1.5) |
| Unavailable | 127 ( 3.0) | 15 ( 1.6) | 49 ( 2.8) | 191 ( 2.7) |
| Number of patients per prescriber, n (%) |  |  |  |  |
| High (≥100 patients) | 2652 (61.8) | 368 (39.2) | 1203 (67.6) | 4223 (60.2) |
| Medium (10-100 patients) | 1087 (25.3) | 281 (29.9) | 332 (18.7) | 1700 (24.2) |
| Low (<10 patients) | 553 (12.9) | 290 (30.9) | 245 (13.8) | 1088 (15.5) |
| DAA regimen, n (%) |  |  |  |  |
| SOF/VEL | 1784 (41.6) | 391 (41.6) | 729 (41.0) | 2904 (41.4) |
| SOF/LDV | 109 ( 2.5) | 84 ( 8.9) | 64 ( 3.6) | 257 ( 3.7) |
| SOF+DCV | 99 ( 2.3) | 92 ( 9.8) | 30 ( 1.7) | 221 ( 3.2) |
| GRZ/ELB | 90 ( 2.1) | 33 ( 3.5) | 44 ( 2.5) | 167 ( 2.4) |
| GLE/PIB | 1199 (27.9) | 222 (23.6) | 742 (41.7) | 2163 (30.9) |
| SOF/VEL/VOX | 883 (20.6) | 68 ( 7.2) | 160 ( 9.0) | 1111 (15.8) |
| Other | 128 ( 3.0) | 49 ( 5.2) | 11 ( 0.6) | 188 ( 2.7) |
| Ribavirin added to DAA regimen, n (%) | 270 ( 6.3) | 57 ( 6.1) | 17 ( 1.0) | 344 ( 4.9) |
| Treatment duration, n (%) |  |  |  |  |
| 8-weeks | 1095 (25.5) | 155 (16.5) | 719 (40.4) | 1969 (28.1) |
| 12-weeks | 3017 (70.3) | 647 (68.9) | 1034 (58.1) | 4698 (67.0) |
| 16-weeks | 93 ( 2.2) | 46 ( 4.9) | 17 ( 1.0) | 156 ( 2.2) |
| 24-weeks | 87 ( 2.0) | 91 ( 9.7) | 10 ( 0.6) | 188 ( 2.7) |
| Discontinued initial treatment, n (%) | 422 ( 9.8) | 449 (47.8) | 835 (46.9) | 1706 (24.3) |

* Retreatment fully dispensed includes individuals receiving a full standard treatment course, extended duration of retreatment and those receiving multiple prescriptions for a standard retreatment course.

^$^ Single dispensation of a single treatment course includes individuals receiving a full course of treatment or retreatment in a single dispensation. These individuals were excluded from the retreatment discontinuation analysis.

**Abbreviations:** SOF/LDV, sofosbuvir/ledipasvir; SOF+DCV, GRZ/ELB, grazoprevir/elbasvir; GLE/PIB, glecaprevir/pibrentasvir; SOF/VEL; sofosbuvir/velpatasvir; SOF/VEL/VOX; sofosbuvir/velpatasvir/voxilaprevir;

^Other includes: sofosbuvir±interferon; paritaprevir/ritonavir/ombitasvir+dasabuvir glecaprevir/pibrentasvir+sofosbuvir; grazoprevir/elbasvir+sofosbuvir, sofosbuvir/ledipasvir+daclatasvir; paritaprevir/ritonavir/ombitasvir+dasabuvir+sofosbuvir

Compared to those with a standard course of retreatment (multiple 28-day repeats) and those that discontinued retreatment, those with a with a single dispensation of a full retreatment course were younger (42 vs. 43 vs. 35 years) and a lower proportion were receiving low-income subsidy prescriptions (50% vs. 67% vs. 37%).

Compared to those with a standard course of treatment and those that discontinued retreatment, a higher proportion of those with a single dispensation of a full treatment course were treated by nurse practitioners (4% vs. 3% vs. 12%) and highly experienced HCV prescribers (62% vs. 39% vs. 68%). A higher proportion received GLE/PIB (28% vs. 24% vs. 42%) and 8-week treatment durations (26% vs. 17% vs. 40%).

The proportion of initial treatment discontinuation among those with a single dispensation of a full retreatment course (47%) was higher than those with a standard course of retreatment (10%), and similar to those that discontinued retreatment (48%).

While some characteristics of this population were indicative of retreatment in prison, others were not. As retreatment is a combination of retreatment for reinfection and treatment failure this adds additional complexity to comparisons. There are high rates of reinfection within prisons (6) and the higher use of GLE/PIB 8-week duration is suggestive of retreatment for reinfection, although this regimen may also be used to retreat treatment failure following discontinuation (7,8). Other factors such as the proportion receiving low-income subsidy prescriptions suggest that dispensation of retreatment as a single course is likely occurring in both prison and community settings.

**Supplementary Table 5. Factors associated with retreatment discontinuation**

|  | Retreatment  fully dispensed (n=4292) | Retreatment discontinued  (n=939) | Unadjusted hazard ratio, 95%CI | p value | Adjusted hazard ratio, 95%CI | p value |
| --- | --- | --- | --- | --- | --- | --- |
| Age in years (median [IQR]) | 42 [32, 52] | 43 [35, 52] | 0.98 (0.93, 1.03)* | <0.001 | 1.05 (0.99, 1.11)* | 0.119 |
| Sex, n (%) |  |  |  |  |  |  |
| Male | 3578 (83.4) | 668 (71.1) | - | - | - | - |
| Female | 714 (16.6) | 271 (28.9) | 1.90 (1.65, 2.19) | <0.001 | 1.36 (1.17, 1.57) | <0.001 |
| Concurrent treatment for HIV, n (%) |  |  |  |  |  |  |
| No | 4194 (97.7) | 917 (97.7) | - | - |  |  |
| Yes | 98 ( 2.3) | 22 ( 2.3) | 0.98 (0.64, 1.49) | 0.910 |  |  |
| Location of patient residence, n (%) |  |  |  |  |  |  |
| Major city | 2580 (60.1) | 620 (66.0) | - | - | - | - |
| Regional | 1504 (35.0) | 282 (30.0) | 0.81 (0.71, 0.94) | 0.004 | 0.87 (0.75, 1.00) | 0.056 |
| Remote | 88 ( 2.1) | 20 ( 2.1) | 0.94 (0.60, 1.47) | 0.784 | 0.84 (0.54, 1.32) | 0.453 |
| Unavailable | 120 ( 2.8) | 17 ( 1.8) | 0.62 (0.38, 0.99) | 0.048 | 0.67 (0.41, 1.09) | 0.109 |
| Prescription payment type, n (%) |  |  |  |  |  |  |
| General | 2161 (50.3) | 306 (32.6) | - | - | - | - |
| Low-income subsidy | 2131 (49.7) | 633 (67.4) | 1.99 (1.73, 2.28) | <0.001 | 1.24 (1.07, 1.43) | 0.004 |
| Year retreatment commenced, n (%) |  |  |  |  |  |  |
| 2017 | 470 (11.0) | 201 (21.4) | - | - | - | - |
| 2018 | 623 (14.5) | 162 (17.3) | 0.83 (0.67, 1.03) | 0.094 | 1.02 (0.80, 1.30) | 0.861 |
| 2019 | 1315 (30.6) | 204 (21.7) | 0.54 (0.44, 0.66) | <0.001 | 0.97 (0.75, 1.26) | 0.827 |
| 2020 | 1061 (24.7) | 201 (21.4) | 0.67 (0.54, 0.82) | <0.001 | 1.27 (0.97, 1.66) | 0.085 |
| 2021 | 823 (19.2) | 171 (18.2) | 0.73 (0.59, 0.90) | 0.004 | 1.22 (0.92, 1.62) | 0.169 |
| Prescriber type, n (%) |  |  |  |  |  |  |
| Gastroenterologist | 1351 (31.5) | 217 (23.1) | - | - | - | - |
| Infectious Diseases Physician | 413 ( 9.6) | 55 ( 5.9) | 0.92 (0.68, 1.24) | 0.938 | 0.93 (0.69, 1.26) | 0.637 |
| General Practitioner | 1885 (43.9) | 566 (60.3) | 1.97 (1.68, 2.31) | <0.001 | 1.26 (1.05, 1.52) | 0.015 |
| General Physician | 169 ( 3.9) | 26 ( 2.8) | 0.96 (0.64, 1.44) | 0.737 | 0.86 (0.57, 1.30) | 0.479 |
| Sexual Physician | 62 ( 1.4) | 9 ( 1.0) | 1.07 (0.55, 2.08) | 0.904 | 1.20 (0.61, 2.36) | 0.588 |
| Psychiatry or addiction specialist | 57 ( 1.3) | 20 ( 2.1) | 2.29 (1.45, 3.62) | <0.001 | 1.39 (0.87, 2.23) | 0.171 |
| Nurse Practitioner | 164 ( 3.8) | 24 ( 2.6) | 1.08 (0.71, 1.65) | 0.727 | 0.99 (0.64, 1.53) | 0.954 |
| Other Specialist | 64 ( 1.5) | 7 ( 0.7) | 0.79 (0.37, 1.68) | 0.538 | 0.77 (0.36, 1.64) | 0.492 |
| Unavailable | 127 ( 3.0) | 15 ( 1.6) | 0.89 (0.53, 1.50) | 0.659 | 1.10 (0.65, 1.87) | 0.725 |
| Number of patients per prescriber, n (%) |  |  |  |  |  |  |
| High (≥100 patients) | 2652 (61.8) | 368 (39.2) | - | - | - | - |
| Medium (10-100 patients) | 1087 (25.3) | 281 (29.9) | 1.71 (1.46, 1.99) | <0.001 | 1.26 (1.07, 1.49) | 0.007 |
| Low (<10 patients) | 553 (12.9) | 290 (30.9) | 3.33 (2.85, 3.88) | <0.001 | 1.99 (1.65, 2.40) | <0.001 |
| DAA regimen, n (%) |  |  |  |  |  |  |
| SOF/VEL | 1784 (41.6) | 391 (41.6) | - | - | - | - |
| SOF/LDV | 109 ( 2.5) | 84 ( 8.9) | 2.41 (1.90, 3.08) | <0.001 | 2.44 (1.87, 3.20) | <0.001 |
| SOF+DCV | 99 ( 2.3) | 92 ( 9.8) | 2.18 (1.72, 2.78) | <0.001 | 1.96 (1.46, 2.64) | <0.001 |
| GRZ/ELB | 90 ( 2.1) | 33 ( 3.5) | 1.41 (0.98, 2.08) | 0.062 | 1.47 (1.01, 2.12) | 0.044 |
| GLE/PIB | 1199 (27.9) | 222 (23.6) | 0.88 (0.75, 1.04) | 0.147 | 1.63 (1.26, 2.12) | <0.001 |
| SOF/VEL/VOX | 883 (20.6) | 68 ( 7.2) | 0.38 (0.29, 0.49) | <0.001 | 0.57 (0.43, 0.75) | <0.001 |
| Other | 128 ( 3.0) | 49 ( 5.2) | 1.27 (0.94, 1.72) | 0.116 | 2.39 (1.67, 3.40) | <0.001 |
| Ribavirin added to DAA regimen, n (%) |  |  |  |  |  |  |
| No | 4022 (93.7) | 882 (93.9) | - | - | - | - |
| Yes | 270 ( 6.3) | 57 ( 6.1) | 0.65 (0.49, 0.85) | 0.002 | 0.65 (0.48, 0.88) | 0.006 |
| Treatment duration, n (%) |  |  |  |  |  |  |
| 8-weeks | 1095 (25.5) | 155 (16.5) | - | - | - | - |
| 12-weeks | 3017 (70.3) | 647 (68.9) | 1.36 (1.14, 1.62) | 0.038 | 2.04 (1.54, 2.70) | <0.001 |
| 16 or 24-weeks | 180 ( 4.2) | 137 (14.6) | 2.02 (1.54, 2.63) | <0.001 | 1.96 (1.42, 2.70) | <0.001 |
| Discontinued initial treatment, n (%) |  |  |  |  |  |  |
| No | 3870 (90.2) | 490 (52.2) | - | - | - | - |
| Yes | 422 ( 9.8) | 449 (47.8) | 5.73 (5.04, 6.51) | <0.001 | 4.41 (3.85, 5.05) | <0.001 |

**Abbreviations:** SOF/LDV, sofosbuvir/ledipasvir; SOF+DCV, GRZ/ELB, grazoprevir/elbasvir; GLE/PIB, glecaprevir/pibrentasvir; SOF/VEL; sofosbuvir/velpatasvir; SOF/VEL/VOX; sofosbuvir/velpatasvir/voxilaprevir;

^Other includes: sofosbuvir±interferon; paritaprevir/ritonavir/ombitasvir+dasabuvir glecaprevir/pibrentasvir+sofosbuvir; grazoprevir/elbasvir+sofosbuvir, sofosbuvir/ledipasvir+daclatasvir; paritaprevir/ritonavir/ombitasvir+dasabuvir+sofosbuvir

* Hazard ratio calculated per ten-year decrease in age

**REFERENCES**

1. Hepatitis C Virus Infection Consensus Statement Working Group. Australian recommendations for the management of hepatitis C virus infection: a consensus statement (September 2018) [Internet]. Melbourne, Australia; 2018. Available from: www.gesa.org.au

2. Hajarizadeh B, Grebely J, Matthews GV, Martinello M, Dore GJ. Uptake of direct-acting antiviral treatment for chronic hepatitis C in Australia. J Viral Hepat. 2018;25(6):640–8.

3. Australian Institute of Health and Welfare. Australia’s welfare 2021 data insights. Canberra; 2021.

4. Hajarizadeh B, Grebely J, Byrne B, Marks P, Amin J, McManus H, Butler T, Cunningham EB, Vickerman P, Martin NK, McHutchison JG, Brainard DM, Treloar C, Chambers GM, Grant L, Mcgrath C, Lloyd AR DG. Evaluation of hepatitis C treatment-as-prevention within an Australian prison prospective cohort: The SToP-C study. Lancet Gastroenterol Hepatol. 2021;

5. Papaluca T, Mcdonald L, Hellard M, Iser D, Papaluca T, Mcdonald L, et al. Outcomes of treatment for hepatitis C in prisoners using a nurse-led , statewide model of care nurse-led , statewide model of care. J Hepatol. 2019;70(5):839–46.

6. Carson JM, Dore GJ, Lloyd AR, Grebely J, Byrne M, Cunningham E, et al. Hepatitis C Virus Reinfection Following Direct-Acting Antiviral Treatment in the Prison Setting: The SToP-C Study. Clinical Infectious Diseases. 2022 Apr 1;ciac246.

7. Carson JM, Hajarizadeh B, Hanson J, O’Beirne J, Iser D, Phillip |, et al. Retreatment for hepatitis C virus direct-acting antiviral therapy virological failure in primary and tertiary settings: The REACH-C cohort. J Viral Hepat. 2022;29:11.

8. Carson JM, Hajarizadeh B, Hanson J, O’Beirne J, Iser D, Read P, et al. Effectiveness of treatment for hepatitis C virus reinfection following direct acting antiviral therapy in the REACH-C cohort. International Journal of Drug Policy. 2021;96:103422.

9. Bernal JL, Cummins S, Gasparrini A. Interrupted time series regression for the evaluation of public health interventions: a tutorial. Int J Epidemiol [Internet]. 2017 Feb 1;46(1):348–55.
